# Low-Cost 3D Printers Enable High-Quality and Automated Sample Preparation and Molecular Detection

**DOI:** 10.1371/journal.pone.0158502

**Published:** 2016-06-30

**Authors:** Kamfai Chan, Mauricio Coen, Justin Hardick, Charlotte A. Gaydos, Kah-Yat Wong, Clayton Smith, Scott A. Wilson, Siva Praneeth Vayugundla, Season Wong

**Affiliations:** 1 AI Biosciences, Inc., College Station, Texas, 77845, United States of America; 2 Division of Infectious Diseases, Department of Medicine, Johns Hopkins University, Baltimore, Maryland, 21205, United States of America; The Scripps Research Institute, UNITED STATES

## Abstract

Most molecular diagnostic assays require upfront sample preparation steps to isolate the target’s nucleic acids, followed by its amplification and detection using various nucleic acid amplification techniques. Because molecular diagnostic methods are generally rather difficult to perform manually without highly trained users, automated and integrated systems are highly desirable but too costly for use at point-of-care or low-resource settings. Here, we showcase the development of a low-cost and rapid nucleic acid isolation and amplification platform by modifying entry-level 3D printers that cost between $400 and $750. Our modifications consisted of replacing the extruder with a tip-comb attachment that houses magnets to conduct magnetic particle-based nucleic acid extraction. We then programmed the 3D printer to conduct motions that can perform high-quality extraction protocols. Up to 12 samples can be processed simultaneously in under 13 minutes and the efficiency of nucleic acid isolation matches well against gold-standard spin-column-based extraction technology. Additionally, we used the 3D printer’s heated bed to supply heat to perform water bath-based polymerase chain reactions (PCRs). Using another attachment to hold PCR tubes, the 3D printer was programmed to automate the process of shuttling PCR tubes between water baths. By eliminating the temperature ramping needed in most commercial thermal cyclers, the run time of a 35-cycle PCR protocol was shortened by 33%. This article demonstrates that for applications in resource-limited settings, expensive nucleic acid extraction devices and thermal cyclers that are used in many central laboratories can be potentially replaced by a device modified from inexpensive entry-level 3D printers.

## Introduction

In recent years, the development of point-of-care (POC) molecular diagnostic devices has become a topic of renewed interest. The resurgence of efforts to develop more reliable, specific, and sensitive POC diagnostic devices has been motivated by an increased interest in solving global public health problems [1–3]. Both proper sample preparation and the availability of such diagnostic devices are critical to the success of reliable molecular diagnostic assays and the subsequent treatment given [4, 5]. Although nucleic acid (NA) purification kits and instruments are available from companies such as Invitrogen, bioMerieux, Roche, and Qiagen, these existing instruments range in cost from $15k-$80k. While manually operated sample preparation methods are also available, they are labor intensive and susceptible to contamination, handling variations, or user errors [6, 7]. Research communities have recognized the need for developing suitable sample preparation technologies, as there are many recent reports published on new NA isolation and purification methods [8–19]. Some have opted to use microfluidic devices to automate preparation steps for POC testing [20–24], but these methods are not yet commercially available at a reasonable price, possibly because of the challenges related to system integration, complexity, manufacturability, and performance reproducibility [25].

The lack of capable devices that are low-cost and can perform high-quality and consistent NA isolation is the primary limiting factor in adapting POC molecular diagnostic tests. Diagnostic tests intended for POC use should focus on being cheaper, more intuitive, and more robust to operate [1, 26–28]. Therefore, there is an urgent need to commercialize an affordable, sensitive, and specific sample preparation device for POC use. A simple, low-cost alternative to conventional lab-based equipment would greatly reduce the barriers to providing modern medical diagnostics to low- and middle-income countries.

Here, we showcase the possibility of leveraging the ever-decreasing price of 3D printers to build a sample preparation device for low resource settings via a magnetic particle (MP) based NA isolation and purification approach. In addition, we also demonstrate the potential to convert a 3D printer into an integrated platform with additional NA amplification capability.

Personal 3D printing for hobbyists and enthusiasts started to grow rapidly in 2011. This sudden expansion of the 3D printing market is partly due to the expiration of a key patent on fused deposition modeling (FDM) developed in the late 1980s, which was then commercialized in 1990 by Stratasys [29]. In FDM, the model or part is produced by precisely extruding a thin line of material (commonly a 0.1–0.3 mm layer thickness) that hardens almost immediately. The outlines created by these lines serve as a base for another layer of material to be extruded on top to give height to the “2D” shape. As tens or hundreds of these layers are extruded, the layers accumulate into a recognizable 3D object. A coiled supply of thermoplastic filament is unreeled so that a filament drive gear can supply material to the extrusion nozzle head (commonly called an extruder). The nozzle head melts the material so that it is able to be extruded. Typically, stepper motors and belts are employed to move the extruder and the printing platform to ensure extrusion at precise coordinates. The movements are controlled by a computer-aided manufacturing software package running on a microcontroller.

After the patent on FDM technology expired, a large open-source development community developed, and both commercial and DIY variants utilizing this type of 3D printer appeared. As a result, the price of this technology has dropped by two orders of magnitude in just a couple of years. Currently, numerous entry-level, low-cost and fully-assembled 3D printers can be purchased for around $400 to $750, which are also improving in terms of print quality, speed, and features. As all 3D printers must have remarkable precision to be in good working order, these low-cost 3D printers do not lack 3D printing quality as the pricing is based on the manufacturer’s business model to charge more for 3D printers capable of printing very large objects (>12 inches in any direction). The mechanics and features of these 3D printers are so versatile that not only can they be used for printing plastics, but they also act as highly precise robotic platforms that can be modified to perform many tasks using open-source software. A few standard features of 3D printers have allowed us to repurpose them for use in automated sample preparation and NA amplification.

Firstly, in 3D printing, printer resolution describes layer thickness and X-Y resolution in micrometers (μm). Typical layer thickness is around 100 μm, although some high-end machines can print layers as thin as 16 μm. The X-Y resolution is comparable to that of laser printers. The printers can also move 60 to 100 mm/second with ease. The speed and resolution control of the 3D printers allow us to control the movements of the printer bed and extruder precisely. By designing a holder for attachments that fits into the extruder location, its movements can be precisely controlled with programming. By mounting the appropriate attachment to this holder, MP-based NA isolation/purification can be accomplished.

Secondly, with the extruder removed from its original location, we can repurpose the extruder’s heater and the temperature control sensor on the extruder to provide controlled heating for sample preparation steps that require elevated temperatures (e.g. lysis and NA elution). Since the temperature of the heated nozzle that deposits plastics such as polylactic acid (PLA) and acrylonitrile butadiene styrene (ABS) typically goes to 185°C for PLA and 230°C for ABS, the heater has the capability to heat an aluminum block to ≥95°C, high enough for DNA denaturation.

Thirdly, 3D printers are often equipped with a heated-bed build platform to improve printing quality by preventing warping. This is because as extruded plastic cools, it shrinks slightly. When this shrinking process does not occur throughout a printed part evenly, the part warps. For printing with PLA and ABS, the aluminum heated bed is maintained at 50–60°C and 100–110°C, respectively. This also means that a heated bed can provide the range of temperatures needed to perform heated incubation, isothermal amplification (e.g., 60°C and under), and even PCR reactions (e.g., 95°C for DNA denaturing).

**Fig 1:**
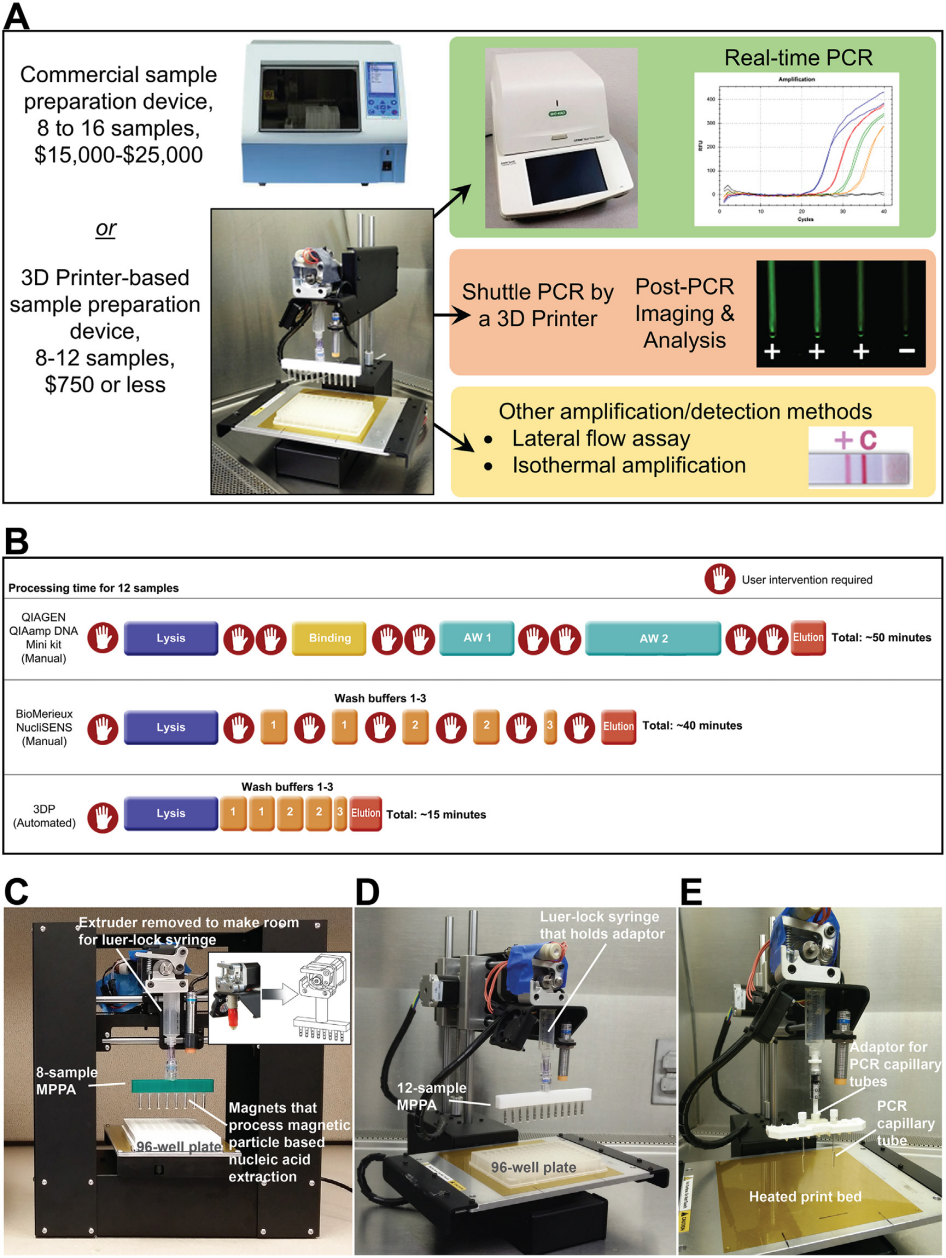
General concept of turning a 3D Printer into a molecular device. We explored the possibility of retrofitting low-cost 3D printers to perform rapid, automated nucleic acid isolation (< 15 min) and amplification (< 25 min). (A) The potential applications of the modified 3D printer in the context of low-cost molecular diagnosis of infectious diseases. Step 1 includes processing varied specimens through magnetic particle based nucleic acid extraction and purification. In step 2, the eluted DNA or RNA can be used in downstream analysis by various molecular detection approaches. Examples are: a) real-time thermal cycling using a commercial thermal cycler (this work), b) probe-based amplification operated by the 3D printer with post-PCR imaging (this work)., c) other molecular detection methods such as a lateral flow assay after PCR or isothermal amplification (underway). (B) Schematic showing the advantages of using the 3D printer for automated extraction. It is faster and requires minimal user-intervention once the process begins. It reduces performance variations induced by users. (C-E) Photos that show the major components that were added to the 3D printer to enable our current work. (C) The Printrbot Play 3D printer (base footprint: 5” × 11”) is the most compact 3D printer we have successfully converted into a nucleic acid extraction device. An 8-sample MPPA (magnetic particle processor attachment) was attached to the luer-lock syringe to carry out magnetic particle based nucleic acid extraction. The magnets were stored inside disposable 0.1-mL PCR tubes to eliminate direct contact between the magnets and samples. (D) The Printrbot Simple 3D printer (base footprint of 11” × 13”) is small enough to fit inside a biosafety cabinet. The 8-sample and 12-sample (shown) MPPA function properly when attached to either the Printrbot Play or Printrbot Simple 3D printer. (E) An adaptor for holding the PCR capillary tubes is attached to the Printrbot Simple 3D printer. The PCR capillary tubes were shuttled by the 3D printer between the 95°C denaturation and the 60°C annealing/extension baths (not shown).

Fig 1A is a schematic showing what the modified 3D printer can perform in the work flow of molecular detection. It can be a rapid and highly efficient nucleic acid extraction device that provides highly purified DNA/RNA for downstream analysis by user-chosen approaches. Alternatively, it has the capability to perform routine 2-step PCR assay. Fig 1B is a schematic showing the labor and time savings of the 3D printer as an extraction device when compared to spin-column and manual magnetic particle-based extraction protocols. There is minimal hands-on time needed once the automated extraction has started. The user will collect the eluted DNA/RNA once the elution step is completed. Shown in Fig 1C–1E are how we repurposed a low-cost 3D printer to perform a NA isolation procedure that is very similar to the workflow used in many MP-based NA isolation methods such as the MagNA Pure Compact Instrument from Roche Life Science. The 3D printer shown in Fig 1C is a $399 fully assembled Printrbot “Play” (Printrbot, Inc., Lincoln, CA). The extruder on the 3D printer was removed and replaced with a 10-mL plastic syringe (Becton Dickson, Franklin Lakes, NJ) with the luer-lock opening pointing down. The back end of the syringe and the flange was cut off to the 8-mL mark of the barre so it could be inserted into the extruder’s mounting slot. The outside of the syringe barrel was sanded down slightly to allow for a tight fit over the extruder’s holding slot. The luer-lock at the syringe allows the user to attach different types of attachments. For example, by attaching a magnetic particle processor attachment (MPPA) that we designed and fabricated with the help of 3D printing (see S1 Fig in Supporting Information), we can conduct NA isolation utilizing the reagents of commercially available MP-based NA isolation kits (described in the materials and methods section). The process is fully automated and no user intervention is needed after sample loading. Eluted NA can be pipetted and tested by any sensing methods, such as paper-based devices, lateral flow assays, or even portable PCR thermal cyclers [1, 27, 30–35]. Another feature of the modified 3D printer is that we can process multiple samples simultaneously by using a standard 96-well plate to store samples and reagents. We also note that the changes we made to the 3D printers were minor and reversible; hence, their 3D printing capability was not lost permanently. The basic operation of the sample preparation steps is shown in S1 Video as a Supporting Information.

In this study, realistic samples such as *Chlamydia trachomatis* in clinically collected urine and dengue virus in human serum were processed using the modified 3D printer. The NA isolation and purification performances were compared with reference methods. We also demonstrate rapid PCR amplification of purified DNA templates using the modified 3D printer. Our platform is not only low-cost, but it also reduces the amount of hands-on-time and it does not require specialized training. Our work showcases the possibility of using an inexpensive device to perform rapid and high quality molecular tests with medium sample throughput.

## Materials and Methods

### Ethics statement

De-identified clinical urine specimens were collected from patients who signed a written consent form from an earlier Johns Hopkins University School of Medicine and Institutional Review Board (IRB) approved study and who agreed to participate in the study and have their de-identified archived sample be utilized in future research for the development of new molecular tests for sexually transmitted disease diagnostics. Original written consent forms were stored in a binder in a locked filing cabinet with access provided to study team coordinator only. Both the consent process and study protocol were written in accordance with the approved guidelines set forth by Johns Hopkins University School of Medicine and Institutional Review Board protocols (IRB numbers: NA_00012998 and NA_00023037).

### Automate extraction and amplification steps using G-code

To repurpose a 3D printer for molecular diagnostics, we needed to determine how to control the device to perform MP-based NA extraction and NA amplification. In 3D printing, printers are often operated using open-source host software such as *Repetier-Host* (Hot-World GmbH & Co., Willich, Germany) and *Cura* (Ultimaker B.V., Geldermalsenhe, the Netherlands) to slice the 3D model drawings and convert them into G-code, the language the 3D printer speaks. The software then either sends the G-code to the printer, or the G-Code can be copied to a Secure Digital card to be used by 3D printers that can be operated without a laptop. In a normal 3D printer, the G-code provides instructions, such as extruder nozzle temperatures, where the tip of the extruder will go, when to deposit melting plastic from the heated nozzle fed with filament, and how much to deposit. We repurposed the 3D printer for NA extraction by writing G-codes to control the motion and many other functions (e.g., controlling temperature at the extruder and the heated bed) of the 3D printer. For example, our G-code can direct the MPPA to move from well to well, and shake at a certain frequency to aid mixing (see S1 Video and S1 Appendix in Supporting Information). While we have successfully programmed four different models of 3D printers to have the capability to perform extraction, we settled on using the *Printrbot Simple* and *Printrbot Play* 3D printers (Printrbot, Inc., Lincoln, California) to perform the work presented in this manuscript.

### Making and using the magnetic particle processor attachment (MPPA)

To perform MP-based extraction, the magnetic particle processor attachment (MPPA) shown in Fig 1C was attached to the luer-lock connector at the syringe barrel. The MPPA shown can process 8 samples using a 96-well plate that holds the samples and reagents. This MPPA’s main body is a 3D-printed piece that has 8 slots (spaced 9 mm apart to match the spacing of the microtiter wells we used for holding reagents for extraction) to accept and tightly hold the caps of 0.1 mL polypropylene PCR tubes (#PCR-0104-C, Axygen Scientific, Union City, CA) made for Qiagen’s Rotor-Gene thermal cycler. Rare-earth permanent magnets (R375-063 and R125-094, Amazing Magnetics LLC, Anaheim, CA) were placed inside each Axygen PCR tube and coupled to the caps already secured inside the MPPA’s main body (see S1 Fig in Supporting Information). The PCR tubes function as the tip-comb seen in some automated sample preparation devices. These rare-earth permanent magnets, shielded by the PCR tubes, were used to collect NA-binding MPs from the lysis solution and transfer MPs into the wells containing washing buffers for the next few steps of NA isolation. The effectiveness of MP collection and transfer leads to superior washing, elution efficiency, and rapid processing. Our MPPA was designed to match the spacing of an 8 × 12 microtiter plate format so that eight samples could be processed simultaneously. Similarly, we also built a 12-tube design to process 12 samples simultaneously in a 96-well plate (Fig 1D). The PCR tubes on the MPPA were replaced after each extraction to avoid cross-contamination between runs. We chose to use commercially available, sterile, disposable PCR plastics to make the tip-comb of MPPA since these items are available inexpensively. A “female luer lock to rotating male luer lock connector” (#20023, Qosina, Ronkonkoma, NY) was then attached to the 3D-printed piece to allow coupling to the luer—lock connector of the syringe. The rotating function of the connector allowed the MPPA to align with the sample wells.

Many semi- or fully-automated devices are sold with pre-filled cartridges for convenience, so the end-users will not have to spend much time to aliquot reagents. In this study, we used sterile polypropylene 96-well plates (0.3 mL per well) to hold the reagents needed for NA extraction. Standard volume plates as well as medium (0.5 mL) and deep well (1 mL) plates can be used on most 3D printer beds since the plate’s standard footprint is only ~128 mm × 86 mm. This gives options to users, should they need to change the sample and wash buffer volume as well as the number of washing steps. A commercially available MP-based NA isolation kit (NucliSENS Magnetic Particle Extraction Kit, bioMerieux, Durham, NC) was purchased and used in this study. Our extraction protocols (e.g., volume, time of incubation, and number of repeated washing steps) were modified from the manufacturer’s suggested protocols. We understand, intuitively, that the resuspension of MPs is needed for high-quality washing, but others [3, 4], including our group in this study, have shown that washing clustered MPs through fluids still provides a high quality of NA in many sample scenarios.

### Enabling the 3D printer to perform heated elution and NA amplification

On a normal 3D printer, an extruder has a heating element and temperature sensor to melt the plastic filament and deposit them precisely using the X, Y, and Z positioning control. After we replaced the extruder with an MPPA, the now detached extruder was repurposed to provide heat to perform tasks such as heated elution or isothermal amplification. A 32-well (8 × 4) aluminum block commonly used for heated dry baths was drilled with a hole large enough to allow the extruder to be tightly inserted (see S2 Fig in Supporting Information). The 3D printer’s host software can be controlled to heat the extruder and the aluminum block to specific temperatures because it has a temperature sensor attached. The aluminum block can be used to perform heated incubation for sample lysis or NA elution during extraction protocols. Although not reported here, the heated block can be used to perform isothermal NA amplifications (e.g., recombinase polymerase amplification) that require incubation temperatures between 40 to 50°C.

Furthermore, we used a 3D printer with a heated printing bed to perform 2-step PCR amplification by transferring reaction tubes between two heated water baths to perform NA denaturation and annealing/extension using a PCR tube holder (Fig 1E). Using the 3D printer’s host software, the heated-bed temperature was set to ~108°C, and two aluminum water baths were placed on the heated bed. The first bath was placed directly on the heated bed to heat the bath and its contents to over 95°C. To create a second water bath for annealing/extension at around 60°C, kitchen-grade aluminum foil was folded to create layers and placed under the aluminum water bath to act as an insulator. The insulating foil reduced the amount of heat transferred to the block, thus stabilizing its temperature as close to the annealing/extension temperature possible (e.g., 60°C). In order to minimize heat loss, the sides of the aluminum bath were insulated by insulating foam.

It is important to point out that both dry heated baths (see Panel A of S3 Fig in Supporting Information) and water baths (Panel B of S3 Fig in Supporting Information) can be utilized for incubation or NA amplification, but the use of a water bath without an individual microtiter well layout (i.e., spaced 9 mm between wells) reduces the need to precisely transport tubes in and out of the blocks and make good contact between the tubes and the block for optimal heat transfer. In addition, water allows for faster heat transfer to the contents inside the PCR tubes. Therefore, the NA amplification steps (especially PCR) can be completed much faster. A water bath setup also accommodates the use of glass capillary PCR tubes, which can further speed up PCR reactions since glass is a better heat conductor. Our setup also does not use a heated lid when PCR is performed. The use of two heated water baths eliminated the temperature ramping needed for the aluminum block used in most commercial thermal cyclers. Therefore, the reagent mix did not spend an excessive amount of time at the denaturation temperature of around 95°C, which would have caused severe evaporation.

### Bacterial culture conditions and clinical samples

*Bacillus subtilis* (TIBS 57, ATCC 31785, ATCC, Manassas, VA) was cultured overnight to the exponential phase in an LB broth (Miller) medium at 37°C. Deidentified clinical urine specimens were provided by Johns Hopkins University School of Medicine. The samples were collected from patients who signed a written consent form to agree to participate in the study and have their samples be utilized in further research and the development of sexually transmitted disease diagnostics. Both the consent process and study protocol were written in accordance with the approved guidelines set forth by Johns Hopkins University School of Medicine and Institutional Review Board protocols (IRB numbers: NA_00012998 and NA_00023037). Samples that were previously confirmed positive for *Chlamydia trachomatis* were used as positive samples. Negative urine samples were those that tested negative for *Chlamydia trachomatis* and were used as negative controls.

### Extraction of nucleic acids using the modified 3D printer

Using the modified 3D printer, NA was extracted with NucliSENS Lysis Buffer and Magnetic Extraction Reagents (bioMerieux, Durham, NC). The following are general steps used in our NA extraction protocols. Prior to extraction, 200 μL of NucliSENS Lysis Buffer and 5 μL of paramagnetic silica beads were added to the second row of wells of a 96-well, round-bottom well plate (Axygen Scientific, Union City, CA). The first row was purposely left empty to reconfirm alignment of the plate with MPPA when the protocol began. Wash Buffer 1 (200 μL) was added to the third and fourth rows of wells. Wash Buffer 2 (200 μL) was added to the fifth and sixth rows of wells. Wash Buffer 3 (200 μL) was added to the seventh row of wells. Elution Buffer (50 μL or 100 μL) was added into the 0.2 mL tubes (BioExpress, Kaysville, UT) and placed in an aluminum block heated to 60°C on the 3D printer heated bed. We chose not to add the elution buffer into the wells of the 96-well plate to avoid having to have an extra step for pipetting the eluate out after elution. When the extraction plate was ready, 100 μL of the sample were added and mixed thoroughly with the Lysis Buffer and paramagnetic silica beads in the second row of wells on the 96-well plate. Sample lysis and NA binding were performed at room temperature for 5 min. NA extraction was then performed automatically by the pre-programmed modified 3D printer. As our work has progressed, we made changes in a few parameters concerning extraction protocols in order to improve the speed and efficiency. For example, elution time was typically 5 min, but it could be shortened to 1 min. From sample addition to the completion of NA elution, the longest extraction protocol we tested was approximately 13 min long (5 min of lysing and NA binding, approximately 3 min of washing steps and 5 min NA elution). Because microtiter plates were used to hold reagents, the type of microtiter plates, volume of reagents, and number of repeated washings can all be modified as needed. This provided flexibility to us and future users in improving NA extraction performance and developing new protocols.

### *B*. *subtilis* cultures processed by 3D printer and real-time PCR of the extracted NA

An overnight culture of *B*. *subtilis* (ATCC 31785) at a concentration of 10^7^ CFU/mL was serially diluted in an LB broth medium. LB broth (100 μL) was added to well A2 of a microtiter plate as a negative control. The 1,000× diluted culture was added to wells B2 and C2. The 100× diluted culture was added to wells D2 and E2. The 10× diluted culture was added to wells F2 and G2. The undiluted culture was added to well H2. DNA extraction was carried out by the modified 3D printer as described above. Elution buffer for each extraction in this experiment was 100 μL. A parallel DNA extraction was also performed manually using the same NucliSENS Lysis Buffer and Magnetic Extraction Reagents (bioMerieux, Durham, NC). Diluted *B*. *subtilis* culture was plated on LB agar plates and incubated overnight at 37°C to estimate the quantity of *B*. *subtilis* added to each well.

Another experiment was done to investigate the sensitivity of our 3D printer extraction protocol. Samples of (100 μL) 10^−1^, 10^−2^, 10^−3^, 10^−4^, and 10^−5^ diluted *B*. *subtilis* culture were added to duplicate wells of the 96-well plate. PCR-grade water (100 μL) was also added to duplicate wells as negative controls. DNA extraction was carried out by the modified 3D printer as described above. Elution buffer for each extraction was 50 μL. Diluted *B*. *subtilis* culture was plated on LB agar plates and incubated overnight at 37°C to estimate the quantity of *B*. *subtilis* added to each well. The 10^−5^ diluted sample had approximately 3×10^2^ CFU/mL, and the original undiluted sample was estimated to have 3×10^7^ CFU/mL.

Real-time PCR targeting *B*. *subtilis* was performed to assess the effectiveness of the modified 3D printer in DNA isolation. In each 20-μL reaction, the PCR mix consisted of 10 μL of the 2× Premix Ex Taq (Clontech Laboratories, Inc., Mountain View, CA), 500 nM of forward primer (Bsub_AP1-F), 500 nM of reverse primer (Bsub_AP1-R), 500 nM of the FAM-labeled hydrolysis probe (BsubAP1), and 2 μL of the DNA template (see Table 1 for primers and probe sequences). The conditions of the real-time PCR were 95°C for 30 seconds, and 40 cycles of 95°C for 12 seconds, and 60°C for 18 seconds, with fluorescence detection carried inside a real-time thermal cycler (Bio-Rad CFX96 Touch, Bio-Rad, Hercules, CA). Real-time PCR results from extractions using 3D printer and manual BioMerieux steps were compared using the quantitation cycle (Cq) value of the reactions.

**Table 1:** Sequence of primers and probes used in this study.

PCR primers / Probes	Sequence	Target	Amplicon Size	Reference
Bsub_AP1-F	5’ TGT AAG CCA TAA GCC ATT CG 3’	*Bacillus subtilis* hypothetical protein	139 bp	[designed internally]
Bsub_AP1-R	5’ GCT ATC ATC CCA ATC TCC GA 3’			
BsubAP1-Probe	5’ FAM-TTC ATG ACC TTC CTC CCG CAC TT-BHQ-1 3’			
CT_292-F	5’ TAG GCG GAT TGA GAG ATT GG 3’	*Chlamydia trachomatis* 16S rRNA gene	165 bp	[37]
CT_456-R	5’ TAT TCC CAA GCG AAA GTG CT 3’			
CT_16S-Probe	5’ FAM-AGA ATC TTT CGC AAT GGA CG-BHQ-1 3’			
D2-F	5’ CAG GCT ATG GCA CYG TCA CGA T 3’	Dengue virus serotype 2 E gene	78 bp	[36]
D2-R	5’ CCA TYT GCA GCA RCA CCA TCT C 3’			
D2-Probe	5’ HEX-CTC YCC RAG AAC GGG CCT CGA CTT CAA-BHQ-1 3’			

### Testing the possibility of cross-contamination/carryover on the 3D printer

To check whether our initial setup was prone to cross-contamination, we carried out *B*. *subtilis* DNA extraction using a very high concentration of cell samples alongside very low-concentration and water-only samples in a 96-well plate. Samples (100 μL each) containing a high concentration of *B*. *subtilis* (10^7^ CFU/extraction well) were pipetted into alternating wells (B1, B3, B5, B7, B9, and B11), followed by pipetting LB broth-only negative controls into wells B2, B4, B6, and B8 and highly diluted culture samples (10 CFU/well) into wells B10 and B12. Because we had 12 samples placed in a single row, a 12-sample MPPA was used. Extraction was performed, and 2 μL from the 50 μL eluted *B*. *subtilis* NA was checked by real-time PCR using the same reaction protocol described in the previous section. The Cq of the reactions from these samples was compared to see if any DNA from highly concentrated samples got into the neighboring wells. Because we used the same 3D printers repeatedly in this study for NA extraction and PCR amplification (although to a lesser extent), we can also estimate if the presence of carry-over contaminants was found in our 3D printer platform by monitoring and comparing the Cq values of the negative controls and the no template controls (NTCs).

### Urine samples processed by 3D printer and characterized by real-time PCR

After completing extractions using cell culture samples, we moved on to test its extraction performance using clinically collected samples. The aim was to show the potential of the 3D printer as a component of an infectious disease diagnostic system. Urine samples confirmed with the presence of *Chlamydia trachomatis* were used as undiluted, 10×, 100×, and 1,000× diluted samples (with duplicate) on a single 96-well plate. The extraction protocol described above was used. The urine sample input was 100 μL and elution output was 50 μL. In order to benchmark the 3D printer’s performance in NA isolation, parallel DNA extraction using commercial spin-column NA isolation kits (QIAamp DNA Mini Kit, Qiagen, Valencia, CA) was performed as well. In both methods, the same input and output volume was used in each extraction.

Real-time PCR was performed by the Bio-Rad CFX96 Touch real-time thermal cycler (Bio-Rad, Hercules, CA) to assess the effectiveness of the modified 3D printer to extract DNA from urine samples. The 20-μL PCR mix consisted of 10 μL of the 2× Premix Ex Taq (Clonetech, Mountain View, CA), 500 nM of forward primer (CT_292-F), 500 nM of reverse primer (CT_456-R), 500 nM of the FAM-labeled hydrolysis probe (CT_16S), and 2 μL of the DNA template (see Table 1 for primers and probe sequences). The run protocol for the real-time PCR was 95°C for 30 seconds, then 40 cycles of 95°C for 5 seconds, and 60°C for 15 seconds, with fluorescence detection carried out. The protocol took 40 min to complete.

Differences between the 3D printer method and the Qiagen spin-column method were examined. Cq values from both methods were analyzed using a paired *t*-test with an online software (GraphPad, La Jolla, CA). A P-value less than 0.05 was considered statistically significant.

### Dengue virus samples processed by a 3D printer and characterized by real-time reverse transcription PCR (real-time RT-PCR)

In addition to the extraction of DNA, we conducted experiments to demonstrate the effective extraction of ribonucleic acid (RNA) for RNA-based pathogen molecular detection. The template we used came from the dengue virus-spiked human serum provided in the CDC DENV-1-4 Real-Time RT-PCR Assay kit obtained from the CDC Dengue Branch. Dengue virus positive control mix (DENV-1-4 +ve control), which consists of heat-inactivated noninfectious DENV-1 Haw, DENV-2 NGC, DENV-3 H87, and DENV-4 H241 serotypes [36], was also diluted 10× and 100× with the Human Specimen Control (HSC). HSC is a noninfectious cultured human cell material suspended in 0.01 M PBS (pH 7.2–7.4) provided in the kit. For each extraction, the sample input was 100 μL and elution output was 50 μL. NA from undiluted DENV-1-4 +ve control, 10× dilution, 100× dilution, and HSC (as negative control) samples were extracted in duplicate wells using the modified 3D printer (8 total samples per extraction run).

The extracted dengue RNA was checked by real-time RT-PCR using the Bio-Rad CFX96 Touch real-time thermal cycler. SuperScript III Platinum One-Step qRT-PCR Kit (Life Technologies, Carlsbad, CA) was used according to the instructions provided by the CDC Dengue Branch. The RT-PCR mix targeting DENV-2 NGC consisted of 12.5 μL of the 2× reaction mix, 0.5 μL of the SuperScript III RT/Platinum Taq mix, 1 μM each of primers D2-F and D2-R, 180 nM of the HEX-labeled D2 hydrolysis probe, and 5 μL of the template extracted. The run protocol of the real-time RT-PCR was 50°C for 30 min (cDNA synthesis), 95°C for 2 min, 45 cycles of 95°C for 15 seconds, and 60°C for 1 min.

### PCR amplification performed by a 3D printer

To further demonstrate the versatility of 3D printers, we used one to perform PCR amplification using two heated water baths. The 10-μL PCR mix consisted of 5 μL of the 2× Premix Ex Taq (Clonetech, Mountain View, CA), 500 nM of forward primer (CT_292-F), 500 nM of reverse primer (CT_456-R), 500 nM of the FAM-labeled hydrolysis probe (CT_16S), and 2 μL of the *C*. *trachomatis* DNA template extracted from urine (undiluted, 10× and 100× dilutions), was pipetted into glass capillaries (507203248, Roche LightCycler Capillaries, Fisher Scientific, Pittsburgh, PA). These capillary tubes were then placed into the PCR tube attachment, and secured to the 3D printer via the luer lock (Fig 1E). The 3D printer moved the tubes into the 95°C denaturation bath for 30 s to begin the hot-start process, then stayed there for another 8 s for DNA denaturation before being moved into the 60°C annealing/extension bath for 12 s. The 3D printer then repeated these movements (except the hot-start process) for another 34 cycles. The 35-cycle PCR took 24 min to complete. After the reaction, the capillary tubes were illuminated by blue LEDs (C503B-BCN-CV0Z0462, Cree Inc., Durham, NC) underneath. Photos of the tubes were taken by a smartphone camera with an orange plastic filter placed in front of the lens. The amplicons were also analyzed using gel electrophoresis with 2.2% Lonza FlashGel DNA cassette (57031, Lonza, Walkersville, MD).

## Results and Discussion

### Performance of DNA extraction from serially diluted *B*. *subtilis* cultures

We first tested the functionality and performance of the modified 3D printer for NA isolation and purified using bacterial cells in LB broth. Duplicates of *B*. *subtilis* cultures at 3 different concentrations (10× 100×, 1000× dilutions), along with an undiluted culture and a negative control, were processed in a single run. The undiluted sample was found to have 10^7^ CFU/mL. Therefore, a 100 μL undiluted sample used in each extraction would have contained 10^6^ CFU. In parallel, DNA was also manually extracted from the same samples using the same NucliSENS reagents. The DNA extraction yield from these serially diluted *B*. *subtilis* samples was assessed and found to be as effective as the bioMerieux manual extraction protocol, as shown by real-time PCR and the Cq obtained. Panel A of S4 Fig in the Supporting Information is a real-time plot for the 3D printer-extracted DNA templates. Null Cq values were obtained when a negative controls were processed using both methods. The PCR amplicons with correct size were confirmed by agarose gel electrophoresis (not shown). The real-time curve data was compared to the templates collected by following the manufacturer’s NucliSENS protocol. Panel B of S4 Fig in the Supporting Information shows that the plots of Cq vs cycle number was very similar between the two methods. Cq values between the two methods were within 0.5 cycle in most cases, with the exception of data from the undiluted culture, where human errors during manual extraction likely caused a lower yield. The R^2^ value (0.987) of the plot indicates that extraction efficiency using 3D printer was linear over at least 4 orders of the concentrations tested in a single extraction run. Amplification efficiency of samples prepared by the 3D printer was found to be 104% (slope of -3.23, efficiency = 10^(-1/slope)^ -1) which indicates that our automated process provided an excellent yield of NA with little PCR inhibitors. The total time needed to process 8 samples using the 3D printer was 13 min while 40 min was needed for the manual bioMerieux NucliSENS protocol (a 67.5% reduction). More importantly, user intervention during washing steps is no longer needed with our 3D printer approach.

### Sensitivity study

**Fig 2:**
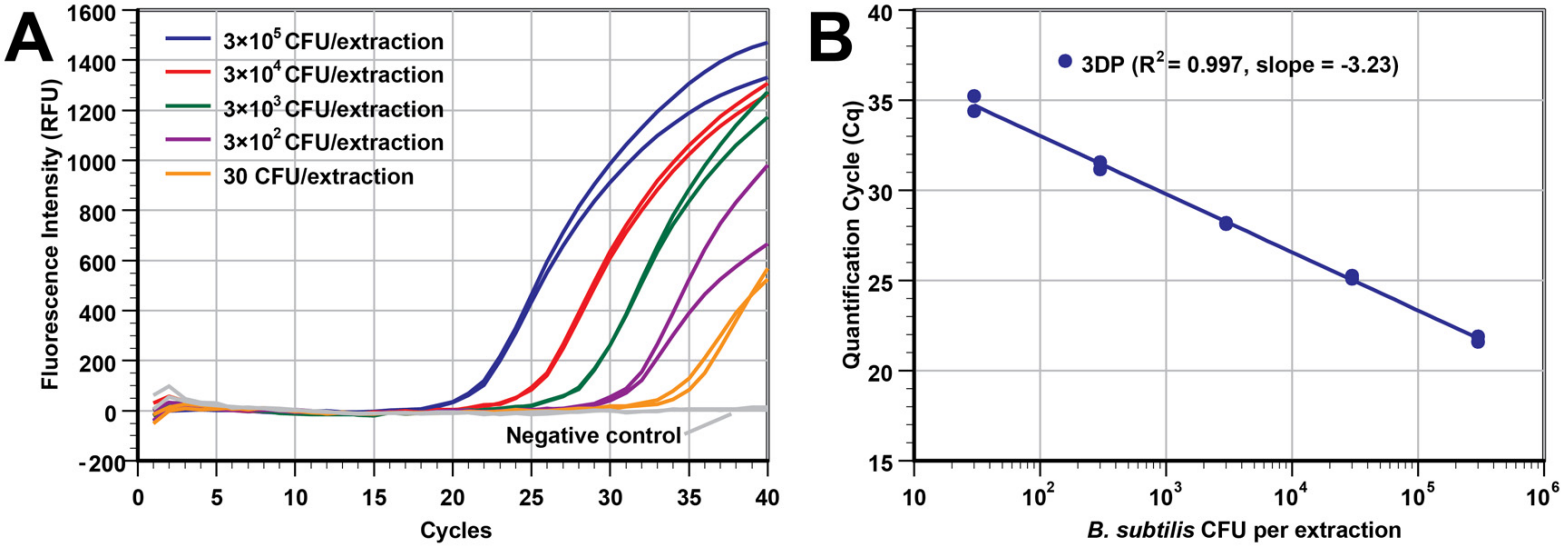
The 3D printer can process samples with a wide range of bacterial concentrations. Different concentrations of *B*. *subtilis* in LB broth medium were extracted using our 3D printer protocol (duplicate wells of 3×10^5^ CFU, 3×10^4^ CFU, 3×10^3^ CFU, 3×10^2^ CFU, 30 CFU, and LB broth as a negative control). DNA was eluted in 50 μL of elution buffer. (A) Real-time PCR curves from the samples with a 5-log difference in concentration. (B) The Cq values of real-time PCR in panel A was plotted against the input concentrations (R^2^ = 0.997, slope = -3.23). The Cq values shown here are slightly better (lower Cq) than those in S4 Fig at the same concentration because we used a smaller elution buffer volume (50 μL instead of 100 μL).

The analytical sensitivity of the extraction by 3D printer was estimated by processing a series of duplicate samples with five different *B*. *subtilis* concentrations and two negative controls (12 total samples in a single run). The cell concentration of the stock solution was 3×10^6^ CFU/mL. Samples containing 3×10^5^ to 30 CFU/extraction were extracted by the 3D printer, with NA eluted into 50 μL of elution buffer. Fig 2A (real-time PCR plot) and Fig 2B (Cq vs concentration) show that the 3D printer was able to handle samples over a wide range of cell concentrations (5 log), and the Cq values show that the yield of extracted DNA was highly correlated to the initial cell concentration, with a R^2^ value of 0.997. By assuming 100% cell lysis and NA extraction, PCR results from the 30 CFU/extraction samples showed that we can theoretically detect 1.2 CFU of *B*. *subtilis* in each PCR reaction when 2 μL of the 50 μL eluted DNA template was used. While not tested, it is possible to extract and detect an even lower cell concentration as judged by the Cq values of the PCR curves at low cell concentrations. The average Cq for the 30 CFU/extraction samples (n = 2) was only 34.81 and the negative control curves (n = 2) were flat with the 40-cycle reaction. Additionally, our extraction approach can offer even higher sensitivity for PCR assays if a larger input volume (currently only at 100 μL) is used in a deep well-plate and larger portion of the eluate is used in each PCR reaction.

### 3D printer vs Qiagen spin-column extraction and quantitative real-time PCR detection of *C*.*trachomatis* in urine

**Fig 3:**
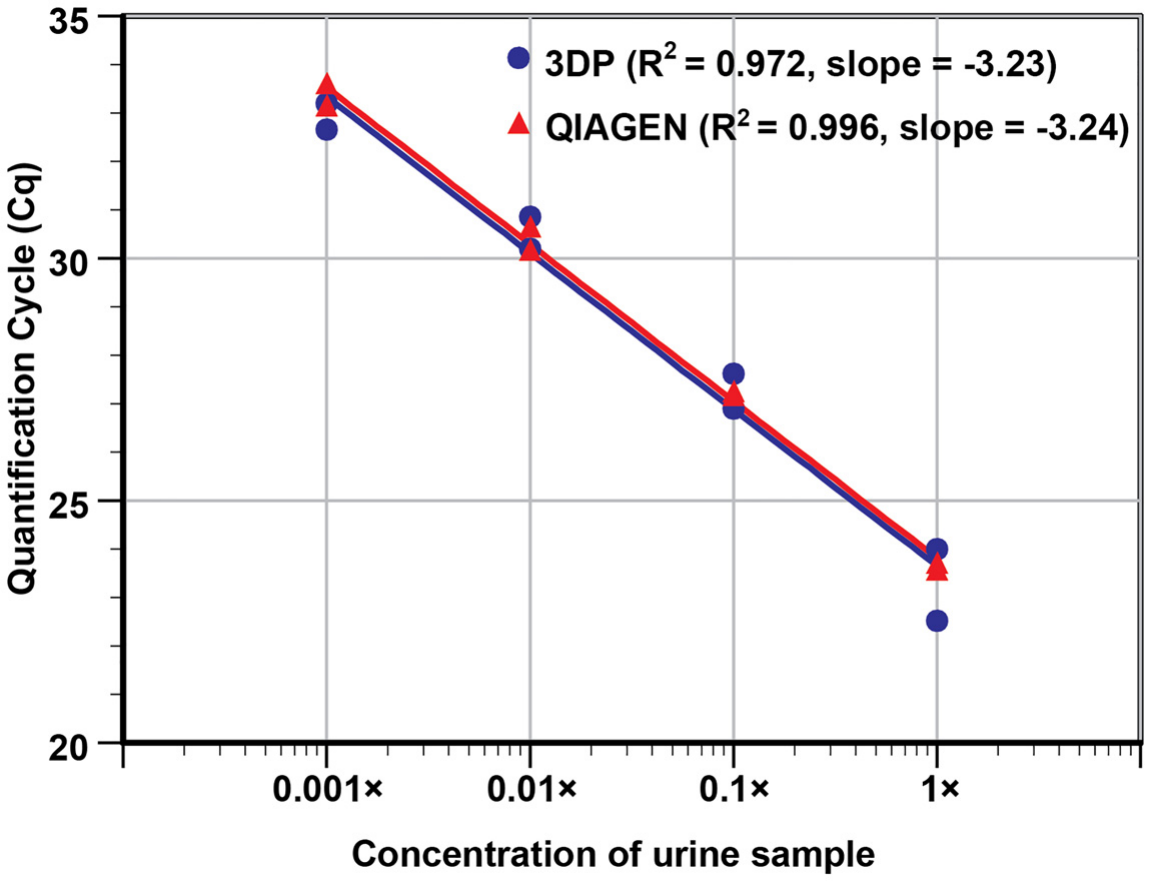
Sensitivity curve for the detection of *C*. *trachomatis* in clinically collected urine. DNA was extracted from undiluted urine, 10×, 100×, and 1,000× diluted urine samples in duplicate, using Qiagen QIAamp spin columns and the 3D printer with MP based DNA extraction. We note that the urine sample input volume, elution output volume, and template volume used in this run were kept identical to allow for proper performance comparison. The R^2^ values (0.972 from the 3D printer vs. 0.996 from the Qiagen column) and the slopes (-3.23 vs. -3.24, respectively) of the two methods listed in the plot indicate that the two extraction approaches performed similarly. With similar Cq values obtained for both methods, a paired t-test P-value of 0.49 further confirms that the 3D printer’s performance in extraction is comparable to one of the gold standards used in nucleic acid isolations.

After successfully testing controlled samples, the performance of the 3D printer extraction on clinical samples was compared to the gold-standard spin-column based extraction method (Qiagen) by performing NA extractions of 10-fold serial dilutions of *C*. *trachomatis* positive urine sample (undiluted to 1,000× diluted). Duplicate samples at each dilution were extracted using both extraction methods and the Cq values from the real-time PCR of the templates were analyzed. Both extraction methods produced similar Cq values at each dilution (Fig 3). The 1,000× diluted *C*. *trachomatis* positive urine samples were detected by both methods and gave Cq values of ~33. NTCs showed a null Cq value. The result suggests that the 3D printer platform potentially can be effective as an upfront sample preparation device for sexually transmitted infections diagnostics.

Based on the slope of the plot (-3.23), the amplification efficiency of urine samples prepared using the 3D printer platform was found to be 104%, indicating that the NA collected was mostly free of PCR inhibitors, as the presence of significant carryover lysate trapped in the clustered MPs would have impeded amplification and reduce PCR efficiency. By directly comparing the PCR result (R^2^ and slope) using DNA collected from the 3D printer and the commercially available spin-column Qiagen kits, the NA yield from the MP-based 3D printer protocols appears to be comparable to the silica membrane-based commercial kit. The paired *t*-test result also indicates that there was no statistical difference between the two extraction methods (two-sided *P* value = 0.49).

### Cross-contamination and carry-over contamination study

**Fig 4:**
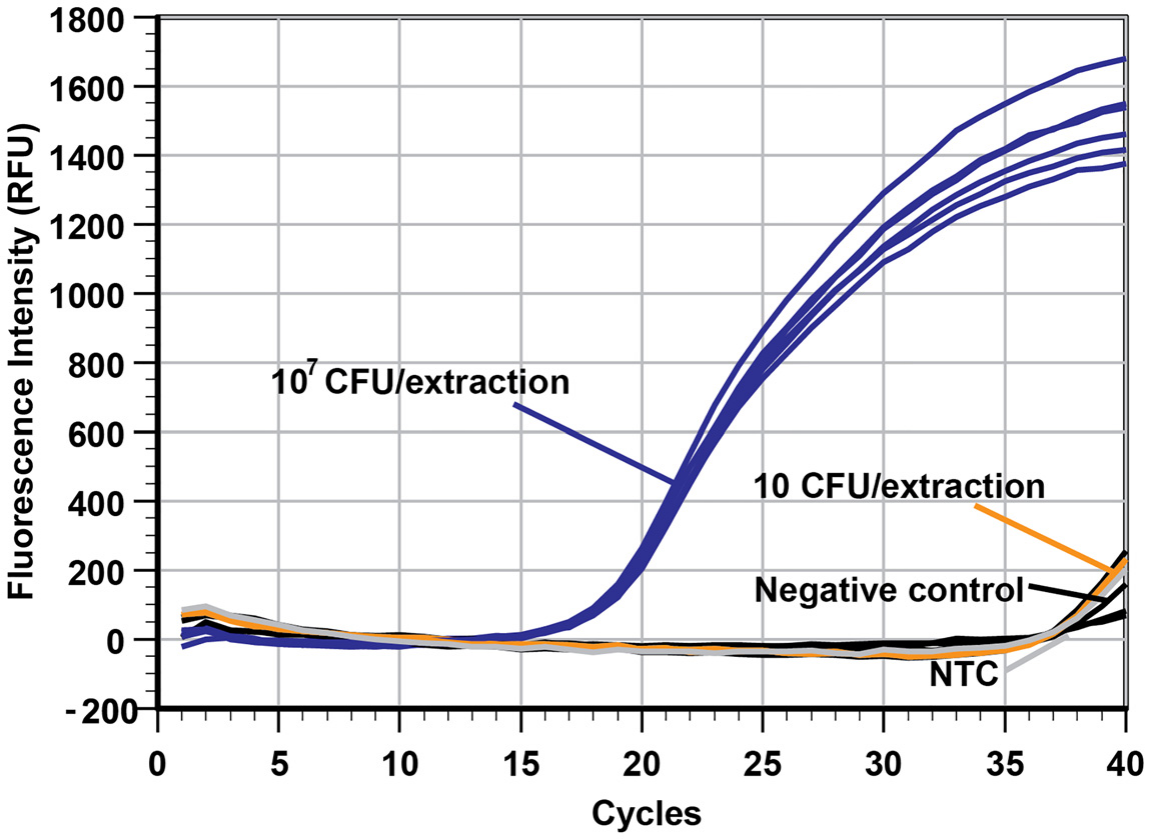
No discernible specimen contamination during 3D printer automated NA extraction. The real-time PCR plot shows that processing a high concentration of cells (10^7^ CFU/extraction) in samples does not lead to cross-contamination in the adjacent wells (only 10 CFU/extraction or negative samples). The NTC is the no template control. In addition, the results suggest that the extraction consistency between wells was very high since the Cq values of the six identical samples at 10^7^ CFU/extraction are almost identical (19.24 ± 0.21, n = 6).

A major concern in the implementation of automated instrumentation to extract NA for use in amplification assays is the potential for cross-contamination of negative specimens from neighboring true positive samples as a consequence of aerosolization. This would lead to false positive diagnostic results. To assess the possibility of cross-contamination between samples adjacent to one another, samples with a very high *B*. *subtilis* concentration (10^7^ CFU/well) were placed immediately next to samples with a very low cell concentration (10 CFU/well) or negative control (LB broth only). The extraction process was carried out, and the results from real-time PCR testing of the templates are shown in Fig 4.

The real-time PCR plot shows that the Cq of each of the 10^7^ CFU/well samples (n = 6) were very similar (Cq ranged from 18.93 to 19.48, average of 19.24 ± 0.21), and the Cq of the two 10 CFU/well samples (39.04 and 39.18) and four negative controls (null, null, 39.85 and 38.93) were not significantly different from that of the NTC reaction (39.44) performed in the same PCR run. This strongly suggests that cross-contamination was not detectable in our system since that would have led to decreased Cq values in the low concentration and negative control samples. To further reduce the risk of cross-contamination, the 3D printer can be housed inside a case to reduce air drafts that may increase the likelihood of contamination by aerosolization.

Additionally, we also observed that carry-over contamination between extraction runs did not occur throughout the study as we often used concentrated samples along with negative control samples. Our real-time PCR results showed null Cq values in majority of the negative control samples. When high Cq values (e.g. 39 and above) were observed with these negative control samples, it was also observed with NTC samples added to the same PCR run. Retesting these samples with fresh PCR reagents showed null Cq value in majority of these negative control and NTC PCR samples, thus confirming that target DNA was rarely in the eluate of neighboring negative samples during this proof-of-concept stage. The high Cq values found in negative control and NTC can be attributed to very small amount of target DNA or amplicons (e.g., 1 copy per PCR) end up being in the PCR master mix when the reagents were prepared.

### Extraction of RNA from dengue virus

**Fig 5:**
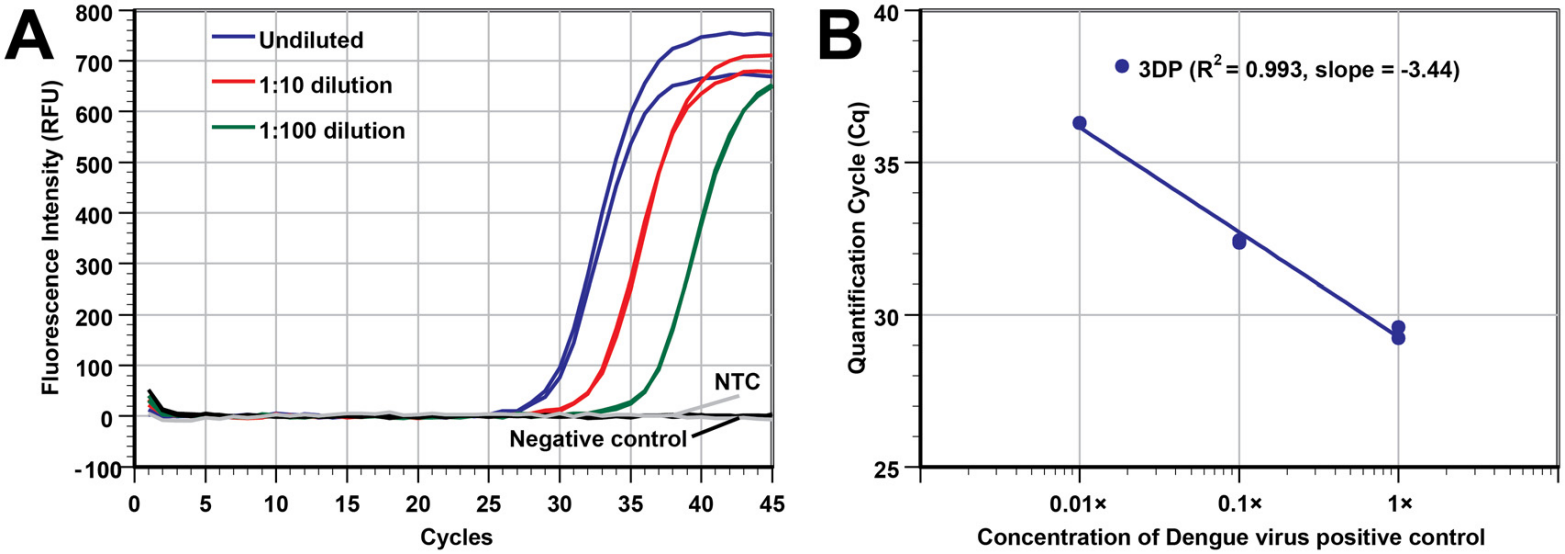
Using the 3D printer to perform RNA extraction. (A) Real-time RT-PCR plot of dengue virus-spiked samples (undiluted sample, 10× and 100× dilutions). Non-infectious cultured human cells suspended in PBS were used as the diluent as well as the negative control. The NTC is the no template control. (B) Plot of Cq vs concentration of viral particles. The slope of the plot (duplicate at each concentration) shows that extraction and PCR were both efficient (slope of -3.44 cycles/log of concentration).

After we successfully demonstrated that the modified 3D printer can isolate and purify DNA effectively, we moved to test its ability to handle extracting RNA targets. A dengue virus positive control was serially diluted (original, 10×, and 100× diluted), and the total NA was extracted by our modified 3D printer. To evaluate the yield of the RNA extracted from Dengue virus, single-plex real-time RT-PCR targeting DENV-2 NGC was performed. Fig 5A shows that the extracted RNA from samples with three-orders of magnitude in viral concentration could be amplified and detected. The Cq values of the duplicates were also close to each other. The R^2^ value (0.993) and the slope -3.44 of the plot in Fig 5B indicate that the extraction efficiency is quite consistent over the 3 orders of magnitude we tested. Also, the negative control sample and the NTC samples had the same Cq (null), which again demonstrates minimal cross-contamination. In short, our modified 3D printer can perform RNA extractions with high performance.

### 3D printer based PCR amplification using *C*. *trachomatis* DNA

**Fig 6:**
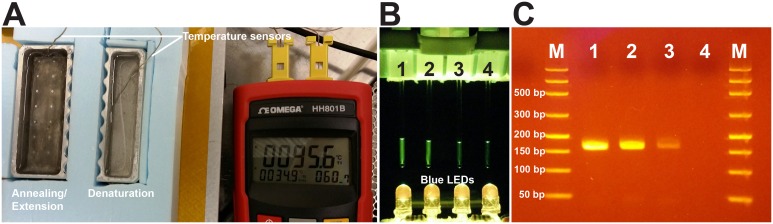
Conducting PCR in aluminum water baths heated by a 3D printer’s heated bed. (A) Top view of the two mini-water baths’ setup. A single heated bed provided the appropriate temperatures (95.6°C and 60.7°C as shown in the data logger) for the NA denaturation and annealing/extension steps needed for PCR. (B) Side view of the setup for capturing fluorescent signal change. Four blue LEDs were placed under the PCR capillary tubes to excite the green fluorescent (FAM) dye-labeled hydrolysis probes inside the tubes. The DNA template used in tube 1 was from undiluted urine sample, tube 2 was 10× diluted urine sample, tube 3 was 100× diluted urine sample, and tube 4 was the NTC. The photo was taken after 35 cycles, by a smartphone camera with an orange plastic filter placed over the lens. The fluorescent signal can also be monitored when the tubes are moved between the water baths by the 3D printer. By viewing the fluorescent intensity of the glass capillary tubes, the user can determine which tubes have positive results and semi-quantitatively determine the template’s concentration if the reactions have not reached the plateau phase of PCR amplification. (C) Gel image from the four tubes shown in panel B. Expected amplicon size is 165 bp. Lane M is DNA ladder (from bottom to top: 50/100/150/200/300/500/800/1500 bp). The intensity of the gel bands corresponds well with the template concentration and the fluorescence from the glass tubes.

We set up the two small water baths that were cut from a 96-well aluminum block with a portion of the interior removed to hold water (Fig 6A). PCR reactions were performed by controlling the 3D printer’s motions. This mimics the archaic method of performing PCR when it was first invented. Both plastic and glass PCR tubes can be used, and PCR results were obtained using the heated bed of a 3D printer as the heat source. While we typically performed real-time PCR for 40 cycles, only a 35-cycle PCR run was used in the experiment presented since running the full 40-cycle would likely lead to saturated fluorescence intensity in the glass tubes and in the gel when the PCRs reached the plateau phase. Fig 6B shows the photos of PCR using glass capillary tubes. The tubes (from left to right) contained a template of extracted DNA from undiluted, 10× diluted, and 100× diluted *C*. *trachomatis* positive urine samples. The last tube on the right was a NTC sample as reference. The signal intensity difference between the glass capillary tubes follows the trend of template concentration. This reaction took only 24 min to complete in the 3D printer while the commercial thermal cycler needed 36 min to complete 35 cycles. Fig 6C shows the gel data from the samples collected from the four capillary tubes imaged in Fig 6B. As expected, the highest band intensity came from the tube that used DNA template extracted from undiluted urine sample. With 10× diluted urine sample, PCR amplicon gave a slightly lower band intensity. For PCR using DNA template extracted from 100× diluted urine sample, gel electrophoresis showed a very faint but still recognizable band when compared to the NTC sample. The weak gel band intensity for the 100× diluted sample after 35 PCR cycles by the 3D printer was not unexpected based on previously determined Cq of ~33 for the same template amplified by a real-time thermal cycler with sensitive fluorescent detector. In short, the PCR results show that a low-cost 3D printer can also perform relatively rapid 2-step PCR amplification in addition to NA extractions. We acknowledge that this setup will be somewhat limited if multiple PCR runs each with different thermal cycling requirements to be performed in a series. Also, running reverse transcription-PCR for RNA using a single heating source would be challenging since a third water bath (e.g., at 50°C) will be needed. Nevertheless, the 3D printer platform offers a means to perform routine and identical PCR runs without needing a commercial thermal cycler.

## Conclusions

We presented a high performing, automated and programmable sample preparation and NA amplification platform that leverage the high precision and low-cost characteristics of consumer-grade 3D printers. The modified 3D printer moves the MP into different reagents to complete NA isolation and purification. Additionally, it can be programmed to move PCR tubes to perform routine 2-step PCR. It currently can process a maximum of 12 samples per run using 96-well plate. We have shown that the modified 3D printer can automatically process urine and serum samples with a wide range of DNA and RNA-based pathogen concentrations in about 13 minutes. This overall time to complete the extraction process is substantially faster than manual bioMerieux magnetic particle and Qiagen spin-column methods. During initial characterization experiments, we also found no evidence of significant cross-contamination or carryover issues. Work has been underway to further reduce the reagent types and washing steps needed in the protocol so that NA extraction can be completed in less than 10 minutes without compromising yield and quality. As for using a 3D printer to perform basic 2-step NA amplification, we have successfully demonstrated that PCR can be completed faster than a commercial thermal cycler, saving at least 12 minutes from each 35-cycle run. Although preliminary, our work has shown that with a few steps of modification, our 3D printer-based platform potentially can provide an inexpensive, automated, medium-throughput molecular detection platform. Furthermore, we note that the changes we made to the 3D printers are minor enough that they are reversible; hence, printing capability of the 3D printer was not lost. With the rapid influx of new features being added to 3D printers (e.g., tablet/Wi-Fi control, Wi-Fi data transfer, multiple extruders, memory card readers, etc.), 3D printers can become an attractive platform for users to build their own high performing molecular device inexpensively and develop their own molecular assays for use in low-resource settings.

## Supporting Information

S1 Appendix G-code for automated NA extraction. (PDF)

S1 Fig Design of the magnetic particle processor attachment (MPPA). The 8-sample magnetic particle processor attachment (MPPA) shown in Fig 1C was attached to the luer-lock connector at the syringe barrel to operate extraction. The MPPA shown can process 8 samples using a 96-well plate that holds the samples and reagents. This MPPA’s main body is a 3D-printed piece that has 8 slots (spaced 9 mm apart to match the spacing of the microtitler wells) to hold the caps of 0.1 mL polypropylene PCR tubes made for Qiagen’s Rotor-Gene thermal cycler. Rare-earth permanent magnets were placed inside each Axygen PCR tube and coupled to the caps are then secured inside the MPPA’s main body. The PCR tubes function as the tip-comb found in some automated sample preparation devices. These rare-earth permanent magnets, shielded by the PCR tubes, were used to collect NA-binding MPs from the lysis solution and transfer MPs into the wells containing washing buffers for the next few steps of NA isolation. The luer-lock connector allows the MPPA to be rotated and align with the wells of the microtiter plate prior to extraction protocol to be started.
(TIF)

S2 Fig Repurposing a 3D printer extruder to heat an aluminum block. Drilling a large hole into a 32-well aluminum block allows for the 3D printer’s extruder to fit in securely. Once placed inside, the user can control the extruder’s temperature to heat the aluminum block to set incubation temperatures. The block can also be wrapped with heat insulating form to minimize heat loss (not shown). (A) The extruder placed outside of the 32-well aluminum block. (B) The extruder inserted into the aluminum block. This block can be wrapped with heat insulating foam to minimize heat loss (not shown).
(TIF)

S3 Fig Using the 3D printer’s heated printing bed to create a thermal cycler. Regular photos (upper) and infrared thermal images (bottom) show that aluminum blocks and water baths can be maintained at two stable temperatures for 2-step PCR by using the 3D printer’s heated bed as a single heat source. (A) Two aluminum blocks were heated until they reached their necessary temperatures, 60°C (left) and 95°C (right). (B) Two aluminum water baths (fabricated by carving out the interior of an aluminum block) were also heated until they reached their necessary temperatures, 60°C (left) and 95°C (right).
(TIF)

S4 Fig Real-time PCR results of NA extracted by the 3D printer. (A) Real-time PCR plot of 3D printer (3DP) extracted *B*. *subtilis* DNA from different concentrations of bacterial cell input in LB broth medium (1 well of 10^6^ CFU, duplicate wells of 10^5^, 10^4^, and 10^3^ CFU, 1 well of LB broth as a negative control). DNA was eluted in 100 μL of elution buffer. (B) Plot of Cq vs cell concentration. The NA extraction performance of the 3D printer (R^2^ = 0.987, slope = -3.23) is similar to the manual NucliSENS protocol as indicated by the similar Cq values at each concentration. One exception was the 10^6^ CFU results, where human errors during manual NucliSENS extraction likely caused a lower yield (higher Cq). This highlights the disadvantage of manually operated protocols. We note that the input sample volume and elution volume used in both methods were identical to avoid a biased presentation of extraction efficiency.
(TIF)

S1 Video Video of a 3D printer in action carrying out magnetic particle based extraction on a 96-well plate. (AVI)
