# Supplementary material for: Low-Cost 3D Printers Enable High-Quality and Automated Sample Preparation and Molecular Detection
Source: PLoS One. 2016 Jun 30;11(6):e0158502. doi: 10.1371/journal.pone.0158502 (PMC4928953; doi:10.1371/journal.pone.0158502)
Supplement: S1 Appendix — (PDF) [file pone.0158502.s001.pdf]

S1 Appendix. G-code for automated NA extraction.

```
; Printrbot Simple

G21
;;;;;;;;;;;;;;;;;;;;;;;;;;;;;;
G90
G01 X75.50 Y145 Z84 F2500

@pause Please attach MPPA
;;;;;;;;;;;;;;;;;;;;;;;;;;;;;;

;Start program
G90
G01 Z77.5 F2500
M140 S70

;Check alignment row
G91
G01 Z-20
G01 Z16
G01 Y-9

;Lysis (30 sec)
G01 Z-7
G01 F20 Z-9
G4  S30
G01 F2500 Z16

;Start Washing
G01 Y-18

;Wash in buffer 1 (10 sec)
G01 Z-16
G00 Y-0.5
G00 Y1
G00 Y-1
G00 Y1
G00 Y-1
G00 Y1
G00 Y-1
G00 Y1
G00 Y-1
G00 Y1
G00 Y-1
G00 Y1
G00 Y-1
G00 Y1
G00 Y-1
G00 Y1
G00 Y-1
G00 Y1
G00 Y-1
G00 Y1
G00 Y-1
G00 Y1
G00 Y-1
```

```
G00 Y1
G00 Y-1
G00 Y1
G00 Y-1
G00 Y1
G00 Y-1
G00 Y1
G00 Y-1
G00 Y1
G00 Y-1
G00 Y1
G00 Y-1
G00 Y1
G00 Y-1
G00 Y1
G00 Y-1
G00 Y1
G00 Y-1
G00 Y1
G00 Y-1
G00 Y1
G00 Y-1
G00 Y1
G00 Y-1
G00 Y1
G00 Y-1
G00 Y1
G00 Y-1
G00 Y1
G00 Y-1
G00 Y1
G00 Y-1
G00 Y1
G00 Y-1
G00 Y1
G00 Y-1
G00 Y1
G00 Y-1
G00 Y1
G00 Y-1
G00 Y1
G00 Y-1
G00 Y1
G00 Y-1
G00 Y1
G00 Y-1
G00 Y1
G00 Y-1
G00 Y1
G00 Y-1
G00 Y1
G00 Y-1
G00 Y1
G00 Y-1
G00 Y1
G00 Y-1
G00 Y1
```

```
G00 Y-1
G00 Y1
G00 Y-1
G00 Y1
G00 Y-1
G00 Y1
G00 Y-1
G00 Y1
G00 Y-1
G00 Y1
G00 Y-1
G00 Y1
G00 Y-1
G00 Y1
G00 Y-1
G00 Y1
G00 Y-1
G00 Y1
G00 Y-1
G00 Y1
G00 Y-1
G00 Y1
G00 Y-1
G00 Y1
G00 Y-1
G00 Y1
G00 Y-1
G00 Y1
G00 Y-1
G00 Y1
G00 Y-1
G00 Y1
G00 Y-1
G00 Y1
G00 Y-1
G00 Y1
G00 Y-1
G00 Y1
G00 Y-1
G00 Y1
G00 Y-1
G00 Y1
G00 Y-1
G00 Y1
G00 Y-1
G00 Y1
G00 Y-1
G00 Y1
G00 Y-1
G00 Y1
G00 Y-1
G00 Y1
G00 Y-1
G00 Y1
G00 Y-1
G00 Y1
G00 Y-1
```

```
G00 Y1
G00 Y-1
G00 Y1
G00 Y-1
G00 Y1
G00 Y-1
G00 Y1
G00 Y-1
G00 Y1
G00 Y-1
G00 Y1
G00 Y-1
G00 Y1
G00 Y-1
G00 Y1
G00 Y-1
G00 Y1
G00 Y-1
G00 Y1
G00 Y-1
G00 Y1
G00 Y-1
G00 Y1
G00 Y-1
G00 Y1
G00 Y-1
G00 Y1
G00 Y-1
G00 Y1
G00 Y-1
G00 Y1
G00 Y-1
G00 Y1
G00 Y-1
G00 Y1
G00 Y-1
G00 Y1
G00 Y-1
G00 Y1
G00 Y-1
G00 Y1
G00 Y-1
G00 Y1
G00 Y-1
G00 Y1
G00 Y-1
G00 Y1
G00 Y-1
G00 Y1
G00 Y-1
G00 Y1
G00 Y-1
G00 Y1
G00 Y-1
G00 Y1
G00 Y-1
G00 Y1
```

```
G00 Y-1
G00 Y1
G00 Y-1
G00 Y1
G00 Y-1
G00 Y1
G00 Y-1
G00 Y1
G00 Y-1
G00 Y1
G00 Y-1
G00 Y1
G00 Y-1
G00 Y1
G00 Y-1
G00 Y1
G00 Y-1
G00 Y1
G00 Y-1
G00 Y1
G00 Y-1
G00 Y1
G00 Y-1
G00 Y1
G00 Y-1
G00 Y1
G00 Y-1
G00 Y1
G00 Y-1
G00 Y1
G00 Y-1
G00 Y1
G00 Y-1
G00 Y1
G00 Y-1
G00 Y1
G00 Y-1
G00 Y1
G00 Y-1
G00 Y1
G00 Y-1
G00 Y1
G00 Y-1
G00 Y1
G00 Y-1
G00 Y1
G00 Y-1
G00 Y1
G00 Y-1
G00 Y1
G00 Y-1
G00 Y1
G00 Y-1
G00 Y1
G00 Y-1
G00 Y1
G00 Y-1
```

```
G00 Y1
G00 Y-1
G00 Y1
G00 Y-1
G00 Y1
G00 Y-1
G00 Y1
G00 Y-1
G00 Y1
G00 Y-1
G00 Y1
G00 Y-1
G00 Y1
G00 Y-1
G00 Y1
G00 Y-1
G00 Y1
G00 Y-1
G00 Y1
G00 Y-1
G00 Y1
G00 Y-1
G00 Y1
G00 Y-1
G00 Y1
G00 Y-1
G00 Y1
G00 Y-1
G00 Y1
G00 Y-1
G00 Y1
G00 Y-1
G00 Y1
G00 Y-1
G00 Y1
G00 Y-1
G00 Y1
G00 Y-1
G00 Y1
G00 Y-1
G00 Y1
G00 Y-1
G00 Y1
G00 Y-1
G00 Y1
G00 Y-1
G00 Y1
G00 Y-1
G00 Y1
G00 Y-1
G00 Y0.5
G4 S1
G01 Z16
G01 Y-9

;2nd Wash in buffer 1 (10 sec)
G01 Z-16
```

```
G00 Y-0.5
G00 Y1
G00 Y-1
G00 Y1
G00 Y-1
G00 Y1
G00 Y-1
G00 Y1
G00 Y-1
G00 Y1
G00 Y-1
G00 Y1
G00 Y-1
G00 Y1
G00 Y-1
G00 Y1
G00 Y-1
G00 Y1
G00 Y-1
G00 Y1
G00 Y-1
G00 Y1
G00 Y-1
G00 Y1
G00 Y-1
G00 Y1
G00 Y-1
G00 Y1
G00 Y-1
G00 Y1
G00 Y-1
G00 Y1
G00 Y-1
G00 Y1
G00 Y-1
G00 Y1
G00 Y-1
G00 Y1
G00 Y-1
G00 Y1
G00 Y-1
G00 Y1
G00 Y-1
G00 Y1
G00 Y-1
G00 Y1
G00 Y-1
G00 Y1
G00 Y-1
G00 Y1
G00 Y-1
G00 Y1
G00 Y-1
G00 Y1
G00 Y-1
G00 Y1
G00 Y-1
```

```
G00 Y1
G00 Y-1
G00 Y1
G00 Y-1
G00 Y1
G00 Y-1
G00 Y1
G00 Y-1
G00 Y1
G00 Y-1
G00 Y1
G00 Y-1
G00 Y1
G00 Y-1
G00 Y1
G00 Y-1
G00 Y1
G00 Y-1
G00 Y1
G00 Y-1
G00 Y1
G00 Y-1
G00 Y1
G00 Y-1
G00 Y1
G00 Y-1
G00 Y1
G00 Y-1
G00 Y1
G00 Y-1
G00 Y1
G00 Y-1
G00 Y1
G00 Y-1
G00 Y1
G00 Y-1
G00 Y1
G00 Y-1
G00 Y1
G00 Y-1
G00 Y1
G00 Y-1
G00 Y1
G00 Y-1
G00 Y1
G00 Y-1
G00 Y1
G00 Y-1
G00 Y1
G00 Y-1
G00 Y1
G00 Y-1
G00 Y1
G00 Y-1
G00 Y1
G00 Y-1
G00 Y1
```

```
G00 Y-1
G00 Y1
G00 Y-1
G00 Y1
G00 Y-1
G00 Y1
G00 Y-1
G00 Y1
G00 Y-1
G00 Y1
G00 Y-1
G00 Y1
G00 Y-1
G00 Y1
G00 Y-1
G00 Y1
G00 Y-1
G00 Y1
G00 Y-1
G00 Y1
G00 Y-1
G00 Y1
G00 Y-1
G00 Y1
G00 Y-1
G00 Y1
G00 Y-1
G00 Y1
G00 Y-1
G00 Y1
G00 Y-1
G00 Y1
G00 Y-1
G00 Y1
G00 Y-1
G00 Y1
G00 Y-1
G00 Y1
G00 Y-1
G00 Y1
G00 Y-1
G00 Y1
G00 Y-1
G00 Y1
G00 Y-1
G00 Y1
G00 Y-1
G00 Y1
G00 Y-1
G00 Y1
G00 Y-1
G00 Y1
G00 Y-1
G00 Y1
G00 Y-1
G00 Y1
G00 Y-1
```

```
G00 Y1
G00 Y-1
G00 Y1
G00 Y-1
G00 Y1
G00 Y-1
G00 Y1
G00 Y-1
G00 Y1
G00 Y-1
G00 Y1
G00 Y-1
G00 Y1
G00 Y-1
G00 Y1
G00 Y-1
G00 Y1
G00 Y-1
G00 Y1
G00 Y-1
G00 Y1
G00 Y-1
G00 Y1
G00 Y-1
G00 Y1
G00 Y-1
G00 Y1
G00 Y-1
G00 Y1
G00 Y-1
G00 Y1
G00 Y-1
G00 Y1
G00 Y-1
G00 Y1
G00 Y-1
G00 Y1
G00 Y-1
G00 Y1
G00 Y-1
G00 Y1
G00 Y-1
G00 Y1
G00 Y-1
G00 Y1
G00 Y-1
G00 Y1
G00 Y-1
G00 Y1
G00 Y-1
G00 Y1
G00 Y-1
G00 Y1
G00 Y-1
G00 Y1
G00 Y-1
G00 Y1
```

```
G00 Y-1
G00 Y1
G00 Y-1
G00 Y1
G00 Y-1
G00 Y1
G00 Y-1
G00 Y1
G00 Y-1
G00 Y1
G00 Y-1
G00 Y1
G00 Y-1
G00 Y1
G00 Y-1
G00 Y1
G00 Y-1
G00 Y1
G00 Y-1
G00 Y1
G00 Y-1
G00 Y1
G00 Y-1
G00 Y1
G00 Y-1
G00 Y1
G00 Y-1
G00 Y1
G00 Y-1
G00 Y1
G00 Y-1
G00 Y1
G00 Y-1
G00 Y1
G00 Y-1
G00 Y1
G00 Y-1
G00 Y1
G00 Y-1
G00 Y1
G00 Y-1
G00 Y1
G00 Y-1
G00 Y1
G00 Y-1
G00 Y1
G00 Y-1
G00 Y1
G00 Y-1
G00 Y1
G00 Y-1
G00 Y1
G00 Y-1
G00 Y1
G00 Y-1
G00 Y1
G00 Y-1
```

```
G00 Y1
G00 Y-1
G00 Y1
G00 Y-1
G00 Y1
G00 Y-1
G00 Y1
G00 Y-1
G00 Y1
G00 Y-1
G00 Y1
G00 Y-1
G00 Y1
G00 Y-1
G00 Y1
G00 Y-1
G00 Y0.5
G4 S1
G01 Z16
G01 Y-9

;Wash in buffer 2 (10 sec)
G01 Z-16
G00 Y-0.5
G00 Y1
G00 Y-1
G00 Y1
G00 Y-1
G00 Y1
G00 Y-1
G00 Y1
G00 Y-1
G00 Y1
G00 Y-1
G00 Y1
G00 Y-1
G00 Y1
G00 Y-1
G00 Y1
G00 Y-1
G00 Y1
G00 Y-1
G00 Y1
G00 Y-1
G00 Y1
G00 Y-1
G00 Y1
G00 Y-1
G00 Y1
G00 Y-1
G00 Y1
G00 Y-1
G00 Y1
G00 Y-1
G00 Y1
G00 Y-1
G00 Y1
```

```
G00 Y-1
G00 Y1
G00 Y-1
G00 Y1
G00 Y-1
G00 Y1
G00 Y-1
G00 Y1
G00 Y-1
G00 Y1
G00 Y-1
G00 Y1
G00 Y-1
G00 Y1
G00 Y-1
G00 Y1
G00 Y-1
G00 Y1
G00 Y-1
G00 Y1
G00 Y-1
G00 Y1
G00 Y-1
G00 Y1
G00 Y-1
G00 Y1
G00 Y-1
G00 Y1
G00 Y-1
G00 Y1
G00 Y-1
G00 Y1
G00 Y-1
G00 Y1
G00 Y-1
G00 Y1
G00 Y-1
G00 Y1
G00 Y-1
G00 Y1
G00 Y-1
G00 Y1
G00 Y-1
G00 Y1
G00 Y-1
G00 Y1
G00 Y-1
G00 Y1
G00 Y-1
G00 Y1
G00 Y-1
G00 Y1
G00 Y-1
G00 Y1
G00 Y-1
G00 Y1
G00 Y-1
```

```
G00 Y1
G00 Y-1
G00 Y1
G00 Y-1
G00 Y1
G00 Y-1
G00 Y1
G00 Y-1
G00 Y1
G00 Y-1
G00 Y1
G00 Y-1
G00 Y1
G00 Y-1
G00 Y1
G00 Y-1
G00 Y1
G00 Y-1
G00 Y1
G00 Y-1
G00 Y1
G00 Y-1
G00 Y1
G00 Y-1
G00 Y1
G00 Y-1
G00 Y1
G00 Y-1
G00 Y1
G00 Y-1
G00 Y1
G00 Y-1
G00 Y1
G00 Y-1
G00 Y1
G00 Y-1
G00 Y1
G00 Y-1
G00 Y1
G00 Y-1
G00 Y1
G00 Y-1
G00 Y1
G00 Y-1
G00 Y1
G00 Y-1
G00 Y1
G00 Y-1
G00 Y1
G00 Y-1
G00 Y1
G00 Y-1
G00 Y1
G00 Y-1
G00 Y1
G00 Y-1
G00 Y1
```

```
G00 Y-1
G00 Y1
G00 Y-1
G00 Y1
G00 Y-1
G00 Y1
G00 Y-1
G00 Y1
G00 Y-1
G00 Y1
G00 Y-1
G00 Y1
G00 Y-1
G00 Y1
G00 Y-1
G00 Y1
G00 Y-1
G00 Y1
G00 Y-1
G00 Y1
G00 Y-1
G00 Y1
G00 Y-1
G00 Y1
G00 Y-1
G00 Y1
G00 Y-1
G00 Y1
G00 Y-1
G00 Y1
G00 Y-1
G00 Y1
G00 Y-1
G00 Y1
G00 Y-1
G00 Y1
G00 Y-1
G00 Y1
G00 Y-1
G00 Y1
G00 Y-1
G00 Y1
G00 Y-1
G00 Y1
G00 Y-1
G00 Y1
G00 Y-1
G00 Y1
G00 Y-1
G00 Y1
G00 Y-1
G00 Y1
G00 Y-1
G00 Y1
G00 Y-1
G00 Y1
G00 Y-1
```

```
G00 Y1
G00 Y-1
G00 Y1
G00 Y-1
G00 Y1
G00 Y-1
G00 Y1
G00 Y-1
G00 Y1
G00 Y-1
G00 Y1
G00 Y-1
G00 Y1
G00 Y-1
G00 Y1
G00 Y-1
G00 Y1
G00 Y-1
G00 Y1
G00 Y-1
G00 Y1
G00 Y-1
G00 Y1
G00 Y-1
G00 Y1
G00 Y-1
G00 Y1
G00 Y-1
G00 Y1
G00 Y-1
G00 Y1
G00 Y-1
G00 Y1
G00 Y-1
G00 Y1
G00 Y-1
G00 Y1
G00 Y-1
G00 Y1
G00 Y-1
G00 Y1
G00 Y-1
G00 Y1
G00 Y-1
G00 Y1
G00 Y-1
G00 Y1
G00 Y-1
G00 Y1
G00 Y-1
G00 Y1
G00 Y-1
G00 Y1
G00 Y-1
G00 Y1
G00 Y-1
G00 Y1
```

```
G00 Y-1
G00 Y1
G00 Y-1
G00 Y1
G00 Y-1
G00 Y1
G00 Y-1
G00 Y1
G00 Y-1
G00 Y1
G00 Y-1
G00 Y1
G00 Y-1
G00 Y1
G00 Y-1
G00 Y1
G00 Y-1
G00 Y1
G00 Y-1
G00 Y1
G00 Y-1
G00 Y1
G00 Y-1
G00 Y1
G00 Y-1
G00 Y1
G00 Y-1
G00 Y1
G00 Y-1
G00 Y1
G00 Y-1
G00 Y1
G00 Y-1
G00 Y1
G00 Y-1
G00 Y1
G00 Y-1
G00 Y1
G00 Y-1
G00 Y1
G00 Y-1
G00 Y1
G00 Y-1
G00 Y1
G00 Y-1
G00 Y0.5
G4 S1
G01 Z16
G01 Y-9

;2nd Wash in buffer 2 (10 sec)
G01 Z-16
G00 Y-0.5
G00 Y1
G00 Y-1
G00 Y1
G00 Y-1
G00 Y1
G00 Y-1
G00 Y1
G00 Y-1
G00 Y1
G00 Y-1
```

```
G00 Y1
G00 Y-1
G00 Y1
G00 Y-1
G00 Y1
G00 Y-1
G00 Y1
G00 Y-1
G00 Y1
G00 Y-1
G00 Y1
G00 Y-1
G00 Y1
G00 Y-1
G00 Y1
G00 Y-1
G00 Y1
G00 Y-1
G00 Y1
G00 Y-1
G00 Y1
G00 Y-1
G00 Y1
G00 Y-1
G00 Y1
G00 Y-1
G00 Y1
G00 Y-1
G00 Y1
G00 Y-1
G00 Y1
G00 Y-1
G00 Y1
G00 Y-1
G00 Y1
G00 Y-1
G00 Y1
G00 Y-1
G00 Y1
G00 Y-1
G00 Y1
G00 Y-1
G00 Y1
G00 Y-1
G00 Y1
G00 Y-1
G00 Y1
G00 Y-1
G00 Y1
G00 Y-1
G00 Y1
G00 Y-1
G00 Y1
G00 Y-1
G00 Y1
G00 Y-1
G00 Y1
```

```
G00 Y-1
G00 Y1
G00 Y-1
G00 Y1
G00 Y-1
G00 Y1
G00 Y-1
G00 Y1
G00 Y-1
G00 Y1
G00 Y-1
G00 Y1
G00 Y-1
G00 Y1
G00 Y-1
G00 Y1
G00 Y-1
G00 Y1
G00 Y-1
G00 Y1
G00 Y-1
G00 Y1
G00 Y-1
G00 Y1
G00 Y-1
G00 Y1
G00 Y-1
G00 Y1
G00 Y-1
G00 Y1
G00 Y-1
G00 Y1
G00 Y-1
G00 Y1
G00 Y-1
G00 Y1
G00 Y-1
G00 Y1
G00 Y-1
G00 Y1
G00 Y-1
G00 Y1
G00 Y-1
G00 Y1
G00 Y-1
G00 Y1
G00 Y-1
G00 Y1
G00 Y-1
G00 Y1
G00 Y-1
G00 Y1
G00 Y-1
G00 Y1
G00 Y-1
G00 Y1
G00 Y-1
```

```
G00 Y1
G00 Y-1
G00 Y1
G00 Y-1
G00 Y1
G00 Y-1
G00 Y1
G00 Y-1
G00 Y1
G00 Y-1
G00 Y1
G00 Y-1
G00 Y1
G00 Y-1
G00 Y1
G00 Y-1
G00 Y1
G00 Y-1
G00 Y1
G00 Y-1
G00 Y1
G00 Y-1
G00 Y1
G00 Y-1
G00 Y1
G00 Y-1
G00 Y1
G00 Y-1
G00 Y1
G00 Y-1
G00 Y1
G00 Y-1
G00 Y1
G00 Y-1
G00 Y1
G00 Y-1
G00 Y1
G00 Y-1
G00 Y1
G00 Y-1
G00 Y1
G00 Y-1
G00 Y1
G00 Y-1
G00 Y1
G00 Y-1
G00 Y1
G00 Y-1
G00 Y1
G00 Y-1
G00 Y1
G00 Y-1
G00 Y1
G00 Y-1
G00 Y1
G00 Y-1
G00 Y1
```

```
G00 Y-1
G00 Y1
G00 Y-1
G00 Y1
G00 Y-1
G00 Y1
G00 Y-1
G00 Y1
G00 Y-1
G00 Y1
G00 Y-1
G00 Y1
G00 Y-1
G00 Y1
G00 Y-1
G00 Y1
G00 Y-1
G00 Y1
G00 Y-1
G00 Y1
G00 Y-1
G00 Y1
G00 Y-1
G00 Y1
G00 Y-1
G00 Y1
G00 Y-1
G00 Y1
G00 Y-1
G00 Y1
G00 Y-1
G00 Y1
G00 Y-1
G00 Y1
G00 Y-1
G00 Y1
G00 Y-1
G00 Y1
G00 Y-1
G00 Y1
G00 Y-1
G00 Y1
G00 Y-1
G00 Y1
G00 Y-1
G00 Y1
G00 Y-1
G00 Y1
G00 Y-1
G00 Y1
G00 Y-1
G00 Y1
G00 Y-1
G00 Y1
G00 Y-1
G00 Y1
G00 Y-1
```

```
G00 Y1
G00 Y-1
G00 Y1
G00 Y-1
G00 Y1
G00 Y-1
G00 Y1
G00 Y-1
G00 Y1
G00 Y-1
G00 Y1
G00 Y-1
G00 Y1
G00 Y-1
G00 Y1
G00 Y-1
G00 Y1
G00 Y-1
G00 Y1
G00 Y-1
G00 Y1
G00 Y-1
G00 Y1
G00 Y-1
G00 Y1
G00 Y-1
G00 Y1
G00 Y-1
G00 Y1
G00 Y-1
G00 Y1
G00 Y-1
G00 Y1
G00 Y-1
G00 Y1
G00 Y-1
G00 Y1
G00 Y-1
G00 Y1
G00 Y-1
G00 Y1
G00 Y-1
G00 Y1
G00 Y-1
G00 Y1
G00 Y-1
G00 Y1
G00 Y-1
G00 Y1
G00 Y-1
G00 Y1
G00 Y-1
G00 Y1
G00 Y-1
G00 Y1
G00 Y-1
G00 Y1
```

```
G00 Y-1
G00 Y1
G00 Y-1
G00 Y1
G00 Y-1
G00 Y0.5
G4 S1
G01 Z16
G01 Y-9

;Wash in buffer 3 (10 sec)
G01 Z-16
G00 Y-0.5
G00 Y1
G00 Y-1
G00 Y1
G00 Y-1
G00 Y1
G00 Y-1
G00 Y1
G00 Y-1
G00 Y1
G00 Y-1
G00 Y1
G00 Y-1
G00 Y1
G00 Y-1
G00 Y1
G00 Y-1
G00 Y1
G00 Y-1
G00 Y1
G00 Y-1
G00 Y1
G00 Y-1
G00 Y1
G00 Y-1
G00 Y1
G00 Y-1
G00 Y1
G00 Y-1
G00 Y1
G00 Y-1
G00 Y1
G00 Y-1
G00 Y1
G00 Y-1
G00 Y1
G00 Y-1
G00 Y1
G00 Y-1
G00 Y1
G00 Y-1
G00 Y1
G00 Y-1
G00 Y1
G00 Y-1
G00 Y1
G00 Y-1
G00 Y1
G00 Y-1
G00 Y1
G00 Y-1
G00 Y1
G00 Y-1
G00 Y1
G00 Y-1
```

```
G00 Y1
G00 Y-1
G00 Y1
G00 Y-1
G00 Y1
G00 Y-1
G00 Y1
G00 Y-1
G00 Y1
G00 Y-1
G00 Y1
G00 Y-1
G00 Y1
G00 Y-1
G00 Y1
G00 Y-1
G00 Y1
G00 Y-1
G00 Y1
G00 Y-1
G00 Y1
G00 Y-1
G00 Y1
G00 Y-1
G00 Y1
G00 Y-1
G00 Y1
G00 Y-1
G00 Y1
G00 Y-1
G00 Y1
G00 Y-1
G00 Y1
G00 Y-1
G00 Y1
G00 Y-1
G00 Y1
G00 Y-1
G00 Y1
G00 Y-1
G00 Y1
G00 Y-1
G00 Y1
G00 Y-1
G00 Y1
G00 Y-1
G00 Y1
G00 Y-1
G00 Y1
G00 Y-1
G00 Y1
G00 Y-1
G00 Y1
G00 Y-1
G00 Y1
G00 Y-1
G00 Y1
```

```
G00 Y-1
G00 Y1
G00 Y-1
G00 Y1
G00 Y-1
G00 Y1
G00 Y-1
G00 Y1
G00 Y-1
G00 Y1
G00 Y-1
G00 Y1
G00 Y-1
G00 Y1
G00 Y-1
G00 Y1
G00 Y-1
G00 Y1
G00 Y-1
G00 Y1
G00 Y-1
G00 Y1
G00 Y-1
G00 Y1
G00 Y-1
G00 Y1
G00 Y-1
G00 Y1
G00 Y-1
G00 Y1
G00 Y-1
G00 Y1
G00 Y-1
G00 Y1
G00 Y-1
G00 Y1
G00 Y-1
G00 Y1
G00 Y-1
G00 Y1
G00 Y-1
G00 Y1
G00 Y-1
G00 Y1
G00 Y-1
G00 Y1
G00 Y-1
G00 Y1
G00 Y-1
G00 Y1
G00 Y-1
G00 Y1
G00 Y-1
G00 Y1
G00 Y-1
G00 Y1
G00 Y-1
```

```
G00 Y1
G00 Y-1
G00 Y1
G00 Y-1
G00 Y1
G00 Y-1
G00 Y1
G00 Y-1
G00 Y1
G00 Y-1
G00 Y1
G00 Y-1
G00 Y1
G00 Y-1
G00 Y1
G00 Y-1
G00 Y1
G00 Y-1
G00 Y1
G00 Y-1
G00 Y1
G00 Y-1
G00 Y1
G00 Y-1
G00 Y1
G00 Y-1
G00 Y1
G00 Y-1
G00 Y1
G00 Y-1
G00 Y1
G00 Y-1
G00 Y1
G00 Y-1
G00 Y1
G00 Y-1
G00 Y1
G00 Y-1
G00 Y1
G00 Y-1
G00 Y1
G00 Y-1
G00 Y1
G00 Y-1
G00 Y1
G00 Y-1
G00 Y1
G00 Y-1
G00 Y1
G00 Y-1
G00 Y1
G00 Y-1
G00 Y1
G00 Y-1
G00 Y1
G00 Y-1
G00 Y1
```

```
G00 Y-1
G00 Y1
G00 Y-1
G00 Y1
G00 Y-1
G00 Y1
G00 Y-1
G00 Y1
G00 Y-1
G00 Y1
G00 Y-1
G00 Y1
G00 Y-1
G00 Y1
G00 Y-1
G00 Y1
G00 Y-1
G00 Y1
G00 Y-1
G00 Y1
G00 Y-1
G00 Y1
G00 Y-1
G00 Y1
G00 Y-1
G00 Y1
G00 Y-1
G00 Y1
G00 Y-1
G00 Y1
G00 Y-1
G00 Y1
G00 Y-1
G00 Y1
G00 Y-1
G00 Y1
G00 Y-1
G00 Y1
G00 Y-1
G00 Y1
G00 Y-1
G00 Y1
G00 Y-1
G00 Y1
G00 Y-1
G00 Y1
G00 Y-1
G00 Y1
G00 Y-1
G00 Y1
G00 Y-1
G00 Y1
G00 Y-1
G00 Y1
G00 Y-1
G00 Y1
G00 Y-1
```

```
G00 Y1
G00 Y-1
G00 Y1
G00 Y-1
G00 Y1
G00 Y-1
G00 Y1
G00 Y-1
G00 Y1
G00 Y-1
G00 Y1
G00 Y-1
G00 Y1
G00 Y-1
G00 Y1
G00 Y-1
G00 Y1
G00 Y-1
G00 Y1
G00 Y-1
G00 Y1
G00 Y-1
G00 Y1
G00 Y-1
G00 Y1
G00 Y-1
G00 Y1
G00 Y-1
G00 Y0.5
G4 S1
G90
G01 Z95
G01 Y8.50

;Elution (5 min)
G90
G01 Z71
G91
G00 Z1
G00 Z-1
G00 Z1
G00 Z-1
G00 Z1
G00 Z-1
G00 Z1
G00 Z-1
G00 Z1
G00 Z-1
G00 Z1
G00 Z-1
G00 Z1
G00 Z-1
G00 Z1
G00 Z-1
G00 Z1
G00 Z-1
G00 Z1
```

```
G00 Z-1
G00 Z1
G00 Z-1
G00 Z1
G00 Z-1
G00 Z1
G00 Z-1
G00 Z1
G00 Z-1
G00 Z1
G00 Z-1
G00 Z1
G00 Z-1
G00 Z1
G00 Z-1
G00 Z1
G00 Z-1
G00 Z1
G00 Z-1
G00 Z1
G00 Z-1
G00 Z1
G00 Z-1
G00 Z1
G00 Z-1
G00 Z1
G00 Z-1
G00 Z1
G00 Z-1
G00 Z1
G00 Z-1
G00 Z1
G00 Z-1
G00 Z1
G00 Z-1
G00 Z1
G00 Z-1
G00 Z1
G00 Z-1
G00 Z1
G00 Z-1
G00 Z1
G00 Z-1
G00 Z1
G00 Z-1
G00 Z1
G00 Z-1
G00 Z1
G00 Z-1
G00 Z1
G00 Z-1
G00 Z1
G00 Z-1
G00 Z1
G00 Z-1
G00 Z1
G00 Z-1
```

```
G00 Z1
G00 Z-1
G00 Z1
G00 Z-1
G00 Z1
G00 Z-1
G00 Z1
G00 Z-1
G00 Z1
G00 Z-1
G00 Z1
G00 Z-1
G00 Z1
G00 Z-1
G00 Z1
G00 Z-1
G00 Z1
G00 Z-1
G00 Z1
G00 Z-1
G00 Z1
G00 Z-1
G00 Z1
G00 Z-1
G00 Z1
G00 Z-1
G00 Z1
G00 Z-1
G00 Z1
G00 Z-1
G00 Z1
G00 Z-1
G00 Z1
G00 Z-1
G00 Z1
G00 Z-1
G00 Z1
G00 Z-1
G00 Z1
G00 Z-1
G00 Z1
G00 Z-1
G00 Z1
G00 Z-1
G00 Z1
G00 Z-1
G00 Z1
G00 Z-1
G00 Z1
G00 Z-1
G00 Z1
G00 Z-1
G00 Z1
G00 Z-1
G00 Z1
G00 Z-1
G00 Z1
```

```
G00 Z-1
G00 Z1
G00 Z-1
G00 Z1
G00 Z-1
G00 Z1
G00 Z-1
G00 Z1
G00 Z-1
G00 Z1
G00 Z-1
G00 Z1
G00 Z-1
G00 Z1
G00 Z-1
G00 Z1
G00 Z-1
G00 Z1
G00 Z-1
G00 Z1
G00 Z-1
G00 Z1
G00 Z-1
G00 Z1
G00 Z-1
G00 Z1
G00 Z-1
G00 Z1
G00 Z-1
G00 Z1
G00 Z-1
G00 Z1
G00 Z-1
G00 Z1
G00 Z-1
G00 Z1
G00 Z-1
G00 Z1
G00 Z-1
G00 Z1
G00 Z-1
G00 Z1
G00 Z-1
G00 Z1
G00 Z-1
G00 Z1
G00 Z-1

G00 Z1
G00 Z-1
G00 Z1
G00 Z-1
G00 Z1
G00 Z-1
G00 Z1
G00 Z-1
G00 Z1
```

```
G00 Z-1
G00 Z1
G00 Z-1
G00 Z1
G00 Z-1
G00 Z1
G00 Z-1
G00 Z1
G00 Z-1
G00 Z1
G00 Z-1
G00 Z1
G00 Z-1
G00 Z1
G00 Z-1
G00 Z1
G00 Z-1
G00 Z1
G00 Z-1
G00 Z1
G00 Z-1
G00 Z1
G00 Z-1
G00 Z1
G00 Z-1
G00 Z1
G00 Z-1
G00 Z1
G00 Z-1
G00 Z1
G00 Z-1
G00 Z1
G00 Z-1
G00 Z1
G00 Z-1
G00 Z1
G00 Z-1
G00 Z1
G00 Z-1
G00 Z1
G00 Z-1
G00 Z1
G00 Z-1
G00 Z1
G00 Z-1
G00 Z1
G00 Z-1
G00 Z1
G00 Z-1
G00 Z1
G00 Z-1
G00 Z1
G00 Z-1
G00 Z1
G00 Z-1
G00 Z1
G00 Z-1
```

```
G00 Z1
G00 Z-1
G00 Z1
G00 Z-1
G00 Z1
G00 Z-1
G00 Z1
G00 Z-1
G00 Z1
G00 Z-1
G00 Z1
G00 Z-1
G00 Z1
G00 Z-1
G00 Z1
G00 Z-1
G00 Z1
G00 Z-1
G00 Z1
G00 Z-1
G00 Z1
G00 Z-1
G00 Z1
G00 Z-1
G00 Z1
G00 Z-1
G00 Z1
G00 Z-1
G00 Z1
G00 Z-1
G00 Z1
G00 Z-1
G00 Z1
G00 Z-1
G00 Z1
G00 Z-1
G00 Z1
G00 Z-1
G00 Z1
G00 Z-1
G00 Z1
G00 Z-1
G00 Z1
G00 Z-1
G00 Z1
G00 Z-1
G00 Z1
G00 Z-1
G00 Z1
G00 Z-1
G00 Z1
G00 Z-1
G00 Z1
G00 Z-1
G00 Z1
G00 Z-1
G00 Z1
```

```
G00 Z-1
G00 Z1
G00 Z-1
G00 Z1
G00 Z-1
G00 Z1
G00 Z-1
G00 Z1
G00 Z-1
G00 Z1
G00 Z-1
G00 Z1
G00 Z-1
G00 Z1
G00 Z-1
G00 Z1
G00 Z-1
G00 Z1
G00 Z-1
G00 Z1
G00 Z-1
G00 Z1
G00 Z-1
G00 Z1
G00 Z-1
G00 Z1
G00 Z-1
G00 Z1
G00 Z-1
G00 Z1
G00 Z-1
G00 Z1
G00 Z-1
G00 Z1
G00 Z-1
G00 Z1
G00 Z-1
G00 Z1
G00 Z-1
G00 Z1
G00 Z-1
G00 Z1
G00 Z-1
G00 Z1
G00 Z-1
G00 Z1
G00 Z-1
G00 Z1
G00 Z-1
G00 Z1
G00 Z-1
G00 Z1
G00 Z-1
G00 Z1
G00 Z-1
G00 Z1
G00 Z-1
```

```
G00 Z1
G00 Z-1
G00 Z1
G00 Z-1
G00 Z1
G00 Z-1
G00 Z1
G00 Z-1
G00 Z1
G00 Z-1
G00 Z1
G00 Z-1
G00 Z1
G00 Z-1
G00 Z1
G00 Z-1
G00 Z1
G00 Z-1
G00 Z1
G00 Z-1
G00 Z1
G00 Z-1
G00 Z1
G00 Z-1
G00 Z1
G00 Z-1
G00 Z1
G00 Z-1
G00 Z1
G00 Z-1
G00 Z1
G00 Z-1
G00 Z1
G00 Z-1
G00 Z1
G00 Z-1
G00 Z1
G00 Z-1
G00 Z1
G00 Z-1
G00 Z1
G00 Z-1
G00 Z1
G00 Z-1
G00 Z1
G00 Z-1
G00 Z1
G00 Z-1
G00 Z1
G00 Z-1
G00 Z1
G00 Z-1
G00 Z1
G00 Z-1
G00 Z1
G00 Z-1
```

```
G00 Z1
G00 Z-1
G00 Z1
G00 Z-1
G00 Z1
G00 Z-1
G00 Z1
G00 Z-1
G00 Z1
G00 Z-1
G00 Z1
G00 Z-1
G00 Z1
G00 Z-1
G00 Z1
G00 Z-1
G00 Z1
G00 Z-1
G00 Z1
G00 Z-1
G00 Z1
G00 Z-1
G00 Z1
G00 Z-1
G00 Z1
G00 Z-1
G00 Z1
G00 Z-1
G00 Z1
G00 Z-1
G00 Z1
G00 Z-1
G00 Z1
G00 Z-1
G00 Z1
G00 Z-1
G00 Z1
G00 Z-1
G00 Z1
G00 Z-1
G00 Z1
G00 Z-1
G00 Z1
G00 Z-1
G00 Z1
G00 Z-1
G00 Z1
G00 Z-1
G00 Z1
G00 Z-1
G00 Z1
G00 Z-1
G00 Z1
G00 Z-1
G00 Z1
G00 Z-1
G00 Z1
```

```
G00 Z-1
G00 Z1
G00 Z-1
G00 Z1
G00 Z-1
G00 Z1
G00 Z-1
G00 Z1
G00 Z-1
G00 Z1
G00 Z-1
G00 Z1
G00 Z-1
G00 Z1
G00 Z-1
G00 Z1
G00 Z-1
G00 Z1
G00 Z-1
G00 Z1
G00 Z-1
G00 Z1
G00 Z-1
G00 Z1
G00 Z-1
G00 Z1
G00 Z-1
G00 Z1
G00 Z-1
G00 Z1
G00 Z-1
G00 Z1
G00 Z-1
G00 Z1
G00 Z-1
G00 Z1
G00 Z-1
G00 Z1
G00 Z-1
G00 Z1
G00 Z-1
G00 Z1
G00 Z-1
G00 Z1
G00 Z-1
G00 Z1
G00 Z-1
G00 Z1
G00 Z-1
G00 Z1
G00 Z-1
G00 Z1
G00 Z-1
G00 Z1
G00 Z-1
G00 Z1
G00 Z-1
```

```
G00 Z1
G00 Z-1
G00 Z1
G00 Z-1
G00 Z1
G00 Z-1
G00 Z1
G00 Z-1
G00 Z1
G00 Z-1
G00 Z1
G00 Z-1
G00 Z1
G00 Z-1
G00 Z1
G00 Z-1
G00 Z1
G00 Z-1
G00 Z1
G00 Z-1
G00 Z1
G00 Z-1
G00 Z1
G00 Z-1
G00 Z1
G00 Z-1
G00 Z1
G00 Z-1
G00 Z1
G00 Z-1
G00 Z1
G00 Z-1
G00 Z1
G00 Z-1
G00 Z1
G00 Z-1
G00 Z1
G00 Z-1
G00 Z1
G00 Z-1
G00 Z1
G00 Z-1
G00 Z1
G00 Z-1
G00 Z1
G00 Z-1
G00 Z1
G00 Z-1
G00 Z1
G00 Z-1
G00 Z1
G00 Z-1
G00 Z1
G00 Z-1
G00 Z1
G00 Z-1
G00 Z1
```

```
G00 Z-1
G00 Z1
G00 Z-1
G00 Z1
G00 Z-1
G00 Z1
G00 Z-1
G00 Z1
G00 Z-1
G00 Z1
G00 Z-1
G00 Z1
G00 Z-1
G00 Z1
G00 Z-1
G00 Z1
G00 Z-1
G00 Z1
G00 Z-1
G00 Z1
G00 Z-1
G00 Z1
G00 Z-1
G00 Z1
G00 Z-1
G00 Z1
G00 Z-1
G00 Z1
G00 Z-1
G00 Z1
G00 Z-1
G00 Z1
G00 Z-1
G00 Z1
G00 Z-1
G00 Z1
G00 Z-1
G00 Z1
G00 Z-1
G00 Z1
G00 Z-1
G00 Z1
G00 Z-1
G00 Z1
G00 Z-1
G00 Z1
G00 Z-1
G00 Z1
G00 Z-1
G00 Z1
G00 Z-1
G00 Z1
G00 Z-1
G00 Z1
G00 Z-1
G00 Z1
G00 Z-1
```

```
G00 Z1
G00 Z-1
G00 Z1
G00 Z-1
G00 Z1
G00 Z-1
G00 Z1
G00 Z-1
G00 Z1
G00 Z-1
G00 Z1
G00 Z-1
G00 Z1
G00 Z-1
G00 Z1
G00 Z-1
G00 Z1
G00 Z-1
G00 Z1
G00 Z-1
G00 Z1
G00 Z-1
G00 Z1
G00 Z-1
G00 Z1
G00 Z-1
G00 Z1
G00 Z-1
G00 Z1
G00 Z-1
G00 Z1
G00 Z-1
G00 Z1
G00 Z-1
G00 Z1
G00 Z-1
G00 Z1
G00 Z-1
G00 Z1
G00 Z-1
G00 Z1
G00 Z-1
G00 Z1
G00 Z-1
G00 Z1
G00 Z-1
G00 Z1
G00 Z-1
G00 Z1
G00 Z-1
G00 Z1
G00 Z-1
G00 Z1
G00 Z-1
G00 Z1
G00 Z-1
G00 Z1
```

```
G00 Z-1
G00 Z1
G00 Z-1
G00 Z1
G00 Z-1
G00 Z1
G00 Z-1
G00 Z1
G00 Z-1
G00 Z1
G00 Z-1
G00 Z1
G00 Z-1
G00 Z1
G00 Z-1
G00 Z1
G00 Z-1
G00 Z1
G00 Z-1
G00 Z1
G00 Z-1
G00 Z1
G00 Z-1
G00 Z1
G00 Z-1
G00 Z1
G00 Z-1
G00 Z1
G00 Z-1
G00 Z1
G00 Z-1
G00 Z1
G00 Z-1
G00 Z1
G00 Z-1
G00 Z1
G00 Z-1
G00 Z1
G00 Z-1
G00 Z1
G00 Z-1
G00 Z1
G00 Z-1
G00 Z1
G00 Z-1
G00 Z1
G00 Z-1
G00 Z1
G00 Z-1
G00 Z1
G00 Z-1
G00 Z1
G00 Z-1
G00 Z1
G00 Z-1
G00 Z1
G00 Z-1
```

```
G00 Z1
G00 Z-1
G00 Z1
G00 Z-1
G00 Z1
G00 Z-1
G00 Z1
G00 Z-1
G00 Z1
G00 Z-1
G00 Z1
G00 Z-1
G00 Z1
G00 Z-1
G00 Z1
G00 Z-1
G00 Z1
G00 Z-1
G00 Z1
G00 Z-1
G00 Z1
G00 Z-1
G00 Z1
G00 Z-1
G00 Z1
G00 Z-1
G00 Z1
G00 Z-1
G00 Z1
G00 Z-1
G00 Z1
G00 Z-1
G00 Z1
G00 Z-1
G00 Z1
G00 Z-1
G00 Z1
G00 Z-1
G00 Z1
G00 Z-1
G00 Z1
G00 Z-1
G00 Z1
G00 Z-1
G00 Z1
G00 Z-1
G00 Z1
G00 Z-1
G00 Z1
G00 Z-1
G00 Z1
G00 Z-1
G00 Z1
G00 Z-1
G00 Z1
G00 Z-1
G00 Z1
```

```
G00 Z-1
G00 Z1
G00 Z-1
G00 Z1
G00 Z-1
G00 Z1
G00 Z-1
G00 Z1
G00 Z-1
G00 Z1
G00 Z-1
G00 Z1
G00 Z-1
G00 Z1
G00 Z-1
G00 Z1
G00 Z-1
G00 Z1
G00 Z-1
G00 Z1
G00 Z-1
G00 Z1
G00 Z-1
G00 Z1
G00 Z-1
G00 Z1
G00 Z-1
G00 Z1
G00 Z-1
G00 Z1
G00 Z-1
G00 Z1
G00 Z-1
G00 Z1
G00 Z-1
G00 Z1
G00 Z-1
G00 Z1
G00 Z-1
G00 Z1
G00 Z-1
G00 Z1
G00 Z-1
G00 Z1
G00 Z-1
G00 Z1
G00 Z-1
G00 Z1
G00 Z-1
G00 Z1
G00 Z-1
G00 Z1
G00 Z-1
G00 Z1
G00 Z-1
G00 Z1
G00 Z-1
```

```
G00 Z1
G00 Z-1
G00 Z1
G00 Z-1
G00 Z1
G00 Z-1
G00 Z1
G00 Z-1
G00 Z1
G00 Z-1
G00 Z1
G00 Z-1
G00 Z1
G00 Z-1
G00 Z1
G00 Z-1
G00 Z1
G00 Z-1
G00 Z1
G00 Z-1
G00 Z1
G00 Z-1
G00 Z1
G00 Z-1
G00 Z1
G00 Z-1
G00 Z1
G00 Z-1
G4 S1

G90
G01 Z100
;End program
```
