# Supplementary figures and images for: Low-Cost 3D Printers Enable High-Quality and Automated Sample Preparation and Molecular Detection
Source: PLoS One. 2016 Jun 30;11(6):e0158502. doi: 10.1371/journal.pone.0158502 (PMC4928953; doi:10.1371/journal.pone.0158502)

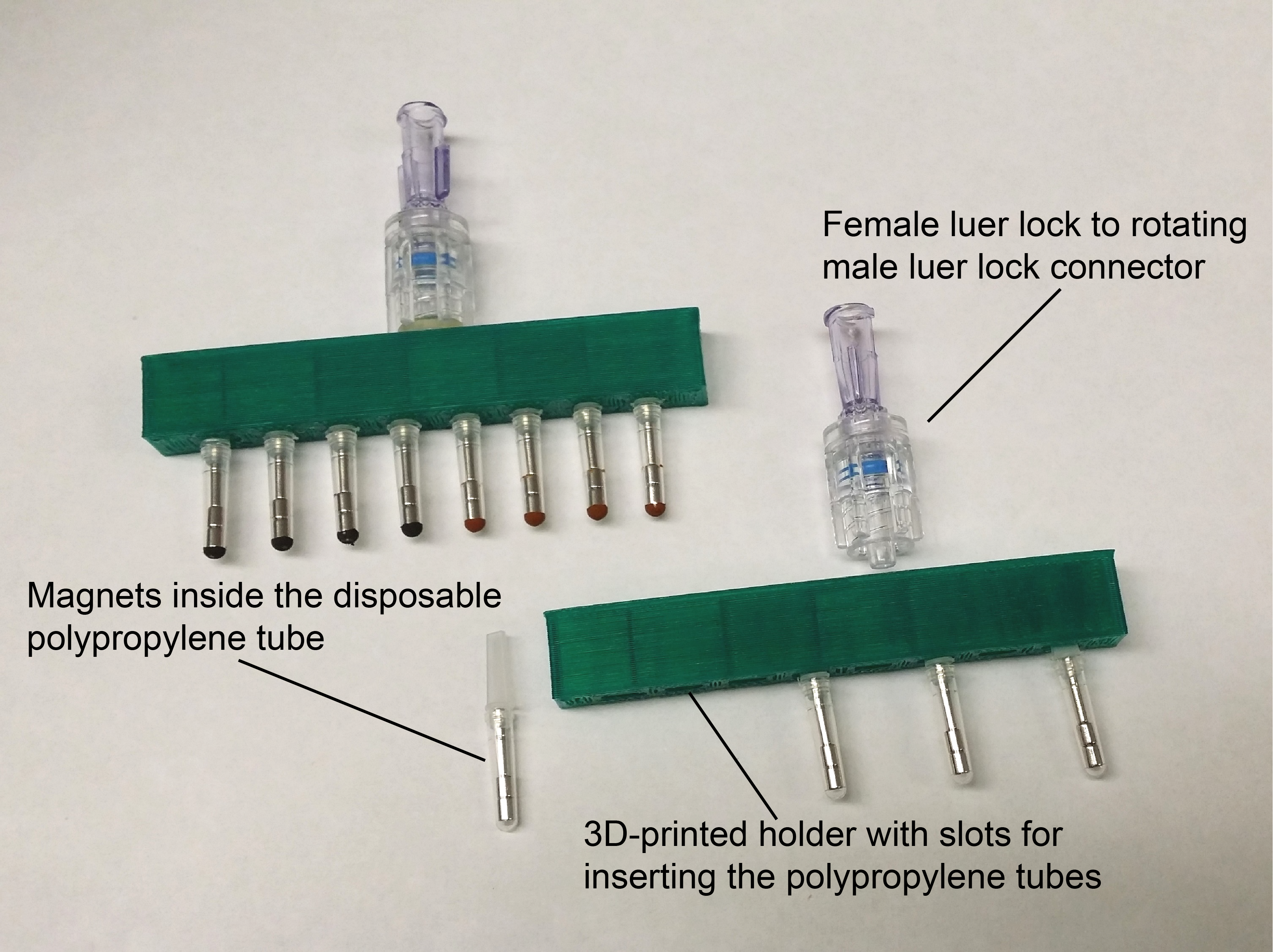

Supplement: S1 Fig — The 8-sample magnetic particle processor attachment (MPPA) shown in Fig 1C was attached to the luer-lock connector at the syringe barrel to operate extraction. The MPPA shown can process 8 samples using a 96-well plate that holds the samples and reagents. This MPPA’s main body is a 3D-printed piece that has 8 slots (spaced 9 mm apart to match the spacing of the microtitler wells) to hold the caps of 0.1 mL polypropylene PCR tubes made for Qiagen’s Rotor-Gene thermal cycler. Rare-earth permanent magnets were placed inside each Axygen PCR tube and coupled to the caps are then secured inside the MPPA’s main body. The PCR tubes function as the tip-comb found in some automated sample preparation devices. These rare-earth permanent magnets, shielded by the PCR tubes, were used to collect NA-binding MPs from the lysis solution and transfer MPs into the wells containing washing buffers for the next few steps of NA isolation. The luer-lock connector allows the MPPA to be rotated and align with the wells of the microtiter plate prior to extraction protocol to be started. (TIF) [file pone.0158502.s002.tif]

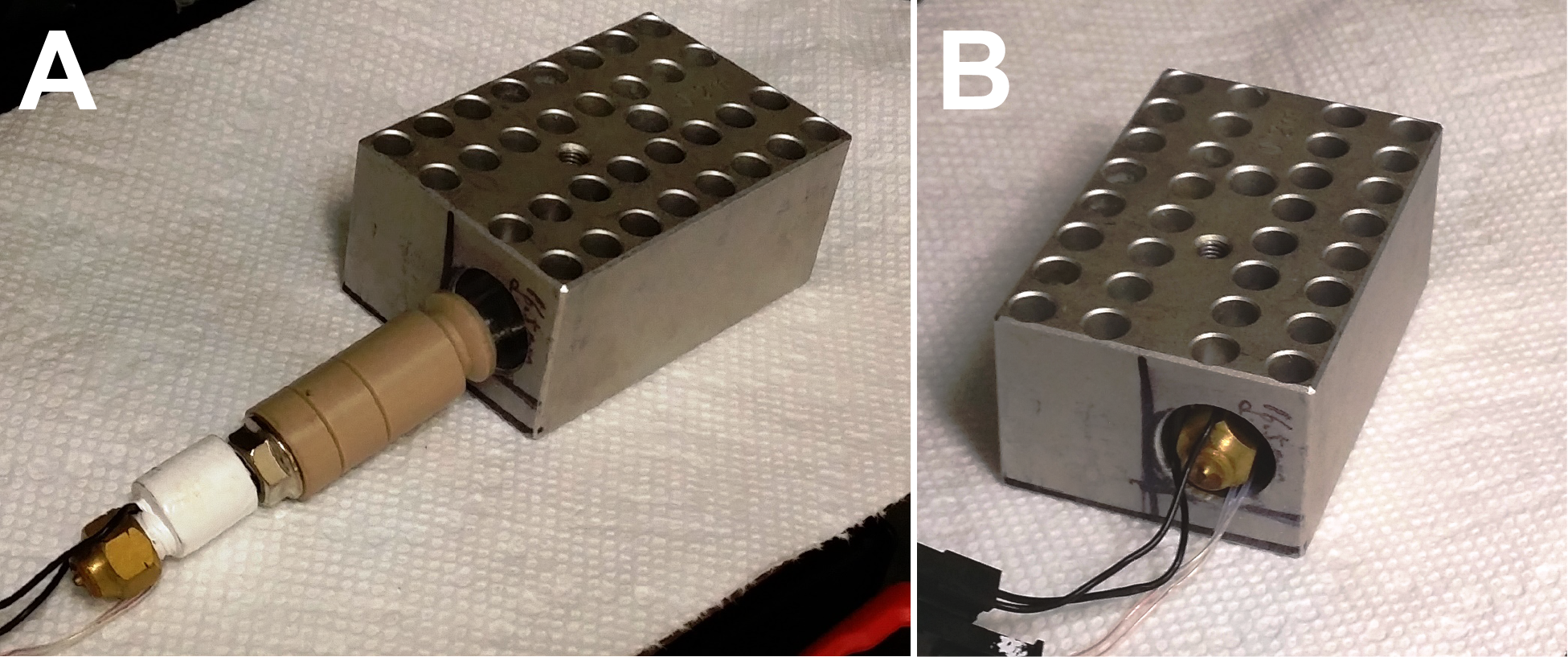

Supplement: S2 Fig — Drilling a large hole into a 32-well aluminum block allows for the 3D printer’s extruder to fit in securely. Once placed inside, the user can control the extruder’s temperature to heat the aluminum block to set incubation temperatures. The block can also be wrapped with heat insulating form to minimize heat loss (not shown). (A) The extruder placed outside of the 32-well aluminum block. (B) The extruder inserted into the aluminum block. This block can be wrapped with heat insulating foam to minimize heat loss (not shown). (TIF) [file pone.0158502.s003.tif]

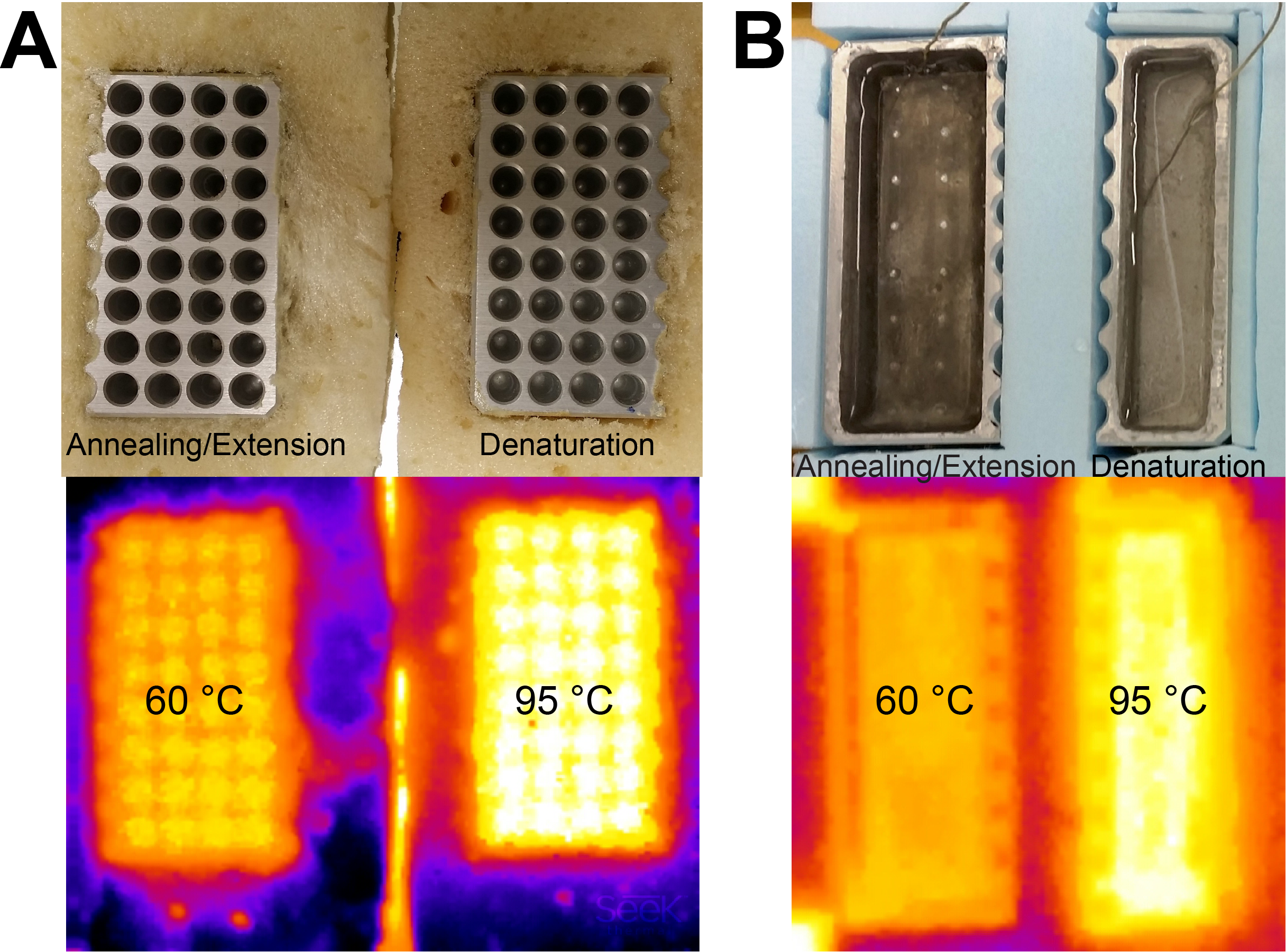

Supplement: S3 Fig — Regular photos (upper) and infrared thermal images (bottom) show that aluminum blocks and water baths can be maintained at two stable temperatures for 2-step PCR by using the 3D printer’s heated bed as a single heat source. (A) Two aluminum blocks were heated until they reached their necessary temperatures, 60°C (left) and 95°C (right). (B) Two aluminum water baths (fabricated by carving out the interior of an aluminum block) were also heated until they reached their necessary temperatures, 60°C (left) and 95°C (right). (TIF) [file pone.0158502.s004.tif]

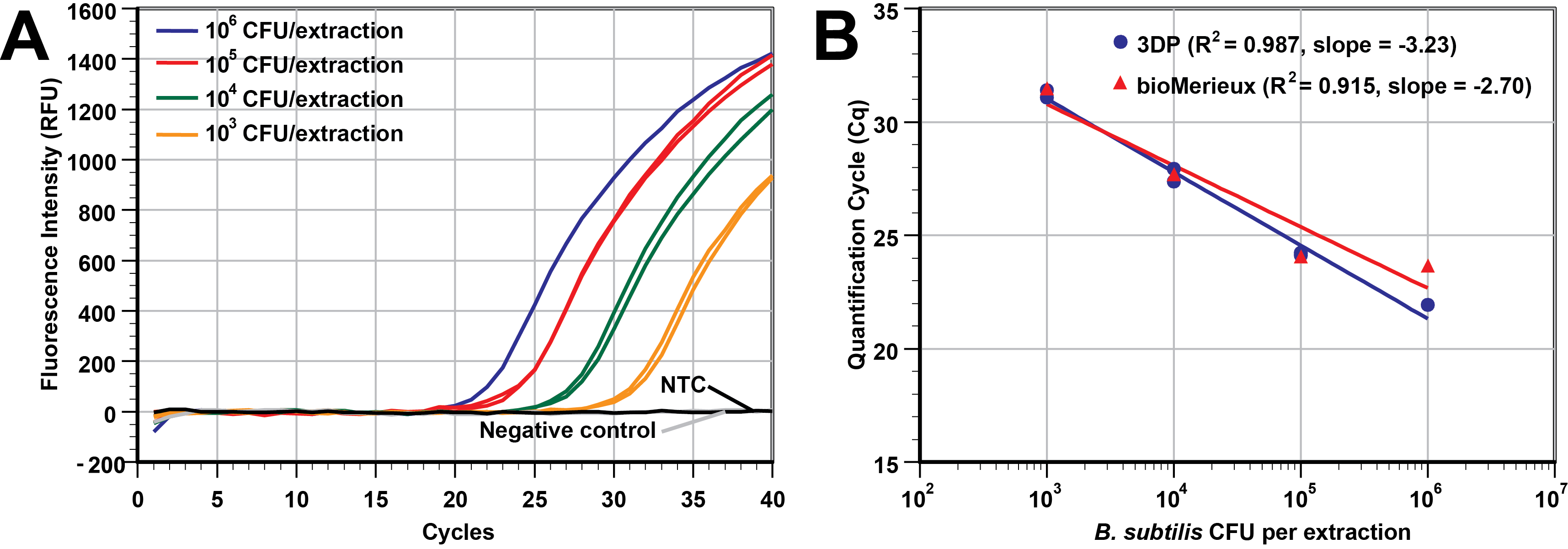

Supplement: S4 Fig — (A) Real-time PCR plot of 3D printer (3DP) extracted B. subtilis DNA from different concentrations of bacterial cell input in LB broth medium (1 well of 106 CFU, duplicate wells of 105, 104, and 103 CFU, 1 well of LB broth as a negative control). DNA was eluted in 100 μL of elution buffer. (B) Plot of Cq vs cell concentration. The NA extraction performance of the 3D printer (R2 = 0.987, slope = -3.23) is similar to the manual NucliSENS protocol as indicated by the similar Cq values at each concentration. One exception was the 106 CFU results, where human errors during manual NucliSENS extraction likely caused a lower yield (higher Cq). This highlights the disadvantage of manually operated protocols. We note that the input sample volume and elution volume used in both methods were identical to avoid a biased presentation of extraction efficiency. (TIF) [file pone.0158502.s005.tif]
